# Contextual centrality: going beyond network structure

**DOI:** 10.1038/s41598-020-62857-4

**Published:** 2020-06-10

**Authors:** Yan Leng, Yehonatan Sella, Rodrigo Ruiz, Alex Pentland

**Affiliations:** 1 Massachusetts Institute of Technology, Cambridge, MA USA; 2 Albert Einstein College of Medicine, New York, USA

**Keywords:** Mathematics and computing, Physics

## Abstract

Centrality is a fundamental network property that ranks nodes by their structural importance. However, the network structure alone may not predict successful diffusion in many applications, such as viral marketing and political campaigns. We propose contextual centrality, which integrates structural positions, the diffusion process, and, most importantly, relevant node characteristics. It nicely generalizes and relates to standard centrality measures. We test the effectiveness of contextual centrality in predicting the eventual outcomes in the adoption of microfinance and weather insurance. Our empirical analysis shows that the contextual centrality of first-informed individuals has higher predictive power than that of other standard centrality measures. Further simulations show that when the diffusion occurs locally, contextual centrality can identify nodes whose local neighborhoods contribute positively. When the diffusion occurs globally, contextual centrality signals whether diffusion may generate negative consequences. Contextual centrality captures more complicated dynamics on networks than traditional centrality measures and has significant implications for network-based interventions.

## Introduction

Individuals, institutions, and industries are increasingly connected in networks where the behavior of one individual entity may generate a global effect^1–3^. Centrality is a fundamental network property that captures an entity’s ability to impact macro processes, such as information diffusion on social networks^1^, cascading failures in financial institutions^3^, and the spreading of market inefficiencies across industries^2^. Many interesting studies have found that the structural positions of individual nodes in a network explain a wide range of behaviors and consequences. Degree centrality predicts who is the first to be infected in a contagion^4^. Eigenvector centrality corresponds to the incentives to maximize social welfare^5^. Katz centrality is proportional to one’s power in strategic interactions in network games^6^. Diffusion centrality depicts an individual’s capability of spreading in information diffusion^7^. These centrality measures operate similarly, aiming to reach a large crowd via diffusion, and are solely dependent on the network structure.

However, several pieces of empirical evidence show that reaching a large crowd may decrease the evaluations of the qualities of the products. For example, sales on Groupon^8^ and public announcements of popular items on Goodreads^9^ are effective strategies in reaching a larger number of customers. However, both studies show that the evaluations of online reviews are negatively affected as a consequence. This phenomenon can be explained by the fact that the increasing popularity will reach individuals who hold negative opinions, and hence, translate into less favorable evaluations of quality. Let us further consider two motivating examples to demonstrate the importance of accounting for the evaluations of the nodes, and more broadly, nodal characteristics.

*Example 1. Viral marketing*. During a viral marketing campaign, the marketing department aims to attract more individuals to adopt the focal product. If we have ex-ante information about the customers’ evaluation of the product or the likelihood of adoption, we can utilize this information to better target individuals who have higher chances of adoption and avoid wasting resources on others.

*Example 2. Political campaign*. Typical Get-Out-The-Vote (GOTV) campaigns include direct mail, phone calls, and social-network advertisement^10,11^. However, rather than simply encouraging every voter to get out the vote, a GOTV strategy should target voters who are more likely to vote for the campaigner’s candidate.

In this paper, we introduce contextual centrality, which builds upon diffusion centrality proposed in Banerjee *et al*. and captures relevant node characteristics in the objective of the diffusion^7,12^. Diffusion centrality focuses on the diffusion process and maximizes the number of individuals who receive the information. In other words, nodes are homogeneous. Contextual centrality is able to integrate the heterogeneity of nodes and aggregate the characteristics over one’s neighborhood; hence it can be used in applications in which reaching different nodes contributes differently to the policy-makers and campaigners. In other words, it generalizes and nests degree, eigenvector, Katz, and diffusion centrality. When the spreadability (the product between the diffusion rate *p* and the largest eigenvalue *λ*_1_ of the adjacency matrix) and the diffusion period *T* are large, contextual centrality linearly scales with eigenvector, Katz, and diffusion centrality. The sign of the scale factor is determined by the joint distribution of nodes’ contributions to the objective of the diffusion and their corresponding structural positions.

We perform an empirical analysis of the diffusion of microfinance and weather insurance showing that the contextual centrality of the first-informed individuals better predicts the adoption decisions than that of the other centrality measures mentioned above. Moreover, simulations on the synthetic data show how network properties and node characteristics collectively influence the performance of different centrality measures. Further, we illustrate the effectiveness of contextual centrality over a wide range of diffusion rates with simulations on the real-world networks and relevant node characteristics in viral marketing and political campaigns.

## Contextual centrality

Given a set of *N* individuals, the adjacency matrix of the network is **A**, an *N*-by-*N* symmetric matrix. The entry *A*_*ij*_ equals 1 if there exists a link between node *i* and node *j*, and 0 otherwise. Let **D** = diag(**d**), where ${d}_{i}={\sum }_{j=1}^{N}{A}_{ij}$ denotes the degree of node *i*. With Singular Value Decomposition, we have **A** = **UΛU**^*T*^, where **Λ** = diag{**Λ**} = {*λ*_1_, *λ*_2_, …, *λ*_*n*_} in a descending order and the corresponding eigenvectors are {**U**_1_, **U**_2_, …, **U**_*n*_} with **U**_1_ being the leading eigenvector. We let ◦ denote the Hadamard product (i.e., element-wise multiplication). We use bold lowercase variables for vectors and bold upper case variables for matrices.

The diffusion process in this paper follows the independent cascade model^13^. It starts with an initial active seed. When node *u* becomes active, it has a single chance to activate each currently inactive neighbor *v* with probability *P*_*uv*_, where ${\bf{P}}\in {{\mathbb{R}}}^{N\times N}$. We follow the terminology by Koschutzki to categorize degree, eigenvector, and Katz centrality as reachability-based centrality measures^14^. Reachability-based centrality measures aim to score a certain node *v* by the expected number of individuals activated if *v* is activated initially, *s*(*v*, **A**, **P**), and hence tend to rank higher the nodes that can reach more nodes in the network. In particular,

1 $$s(v,{\bf{A}},{\bf{P}})=\mathop{\sum }\limits_{i}^{N}{r}_{i}(v,{\bf{A}},{\bf{P}}),$$

where *r*_*i*_(*v*, **A**, **P**) denotes the probability that *i* is activated if *v* is initially activated^13,15,16^. In practice, *s*(*v*, **A**, **P**) is hard to estimate. Different reachability-based centrality measures estimate it in different ways. Diffusion centrality extends and generalizes these standard centrality measures^12^. It operates on the assumption that the activation probability of an individual *i* is correlated with the number of times *i* “hears” the information originating from the individual to be scored. Diffusion centrality measures how extensively the information spreads as a function of the initial node^12^. In other words, diffusion centrality scores node *v* by the expected number of times some piece of information originating from *v* is heard by others within a finite number of time periods *T*, *s*’(*v*, **A**, **P**, *T*),

2 $$s{\prime} (v,{\bf{A}},{\bf{P}},T)=\mathop{\sum }\limits_{i}^{N}{r}_{i}^{{\prime} }(v,{\bf{A}},{\bf{P}},T),$$

where ${r}_{i}^{{\prime} }(v,{\bf{A}},{\bf{P}},T)$ is the expected number of times individual *i* receives the information if *v* is seeded. Equation (2) has at least two advantages over Eq. (1). First, ${r}_{i}^{{\prime} }(v,{\bf{A}},{\bf{P}},T)$ is computationally more efficient than tedious simulations to get *r*_*i*_(*v*, **A**, **P**). Second, it nests degree, eigenvector, and Katz centrality^7^ . It is worth noting that Eqs. (1) and (2) differ in a couple of ways. First, since ${r{\prime} }_{i}(v,{\bf{A}},{\bf{P}},T)$ is the expected number of times *i* hears a piece of information, it may exceed 1. Meanwhile, since ${r}_{i}(v,{\bf{A}},{\bf{P}})$ is the probability that *i* receives the information, it is bounded by 1. Second, in independent cascade model, each activated individual has a single chance to activate the non-activated neighbors. However, with the random walks of information transmission in contextual centrality, each activated individual has multiple chances with decaying probability to activate their neighbors.

Both Eqs. (1) and (2) assume that individuals are homogeneous and contribute equally to the objectives if they have been activated. However, in many real-world scenarios, such as the two examples mentioned above, the payoff for the campaigner does not grow with the size of the cascade. Instead, different nodes contribute differently. Formally, let *y*_*i*_ be the contribution of individual *i* to the cascade payoff upon being activated. Note that *y*_*i*_ is context-dependent and is measured differently in different scenarios. For example, in a market campaign, *y*_*i*_ can be *i*’s likelihood of adoption. In a political campaign, *y*_*i*_ can be the likelihood that *i* votes for the campaigner’s political party. With the independent cascade model, an individual *v* should be scored according to the cascade payoff if *v* is first-activated, *s*_*c*_(*v*, **A**, **p**). With this, we present the following equation as a generalization and extension to Eq. (1) with heterogeneous **y**,

3 $${\rm{cascade}}\,{\rm{payoff}}:\,{s}_{c}(v,{\bf{A}},{\bf{P}})=\mathop{\sum }\limits_{i}^{N}{r}_{i}(v,{\bf{A}},{\bf{P}}){y}_{i}.$$

Similar to diffusion centrality, we score nodes with the following approximated cascade payoff, ${s{\prime} }_{c}(v,{\bf{A}},{\bf{p}},T)$, with heterogeneous **y**,

4 $${\rm{approximated}}\,{\rm{cascade}}\,{\rm{payoff}}:\,{s}_{c}^{{\prime} }(v,{\bf{A}},{\bf{P}},T)=\mathop{\sum }\limits_{i}^{N}{r}_{i}^{{\prime} }(v,{\bf{A}},{\bf{P}},T){y}_{i}.$$

This formulation generalizes diffusion centrality and inherits its nice properties in nesting existing reachability-based centrality measures. Moreover, it is easier to compute than Eq. (3), with this scoring function, we now formally propose contextual centrality.

The computational complexity of the algorithm to score according to Eq. (3) is *O*(*NMT*), where *M* is the average degree, and *T* is the lengths of the paths that have been inspected. Note that the computational complexity of the formulation (5) is *O*(*NMT*). We repeat the operation of multiplying a vector of length *N* with a sparse matrix, which has an average of *M* entries per row for *T* times. This significantly reduces the run time.

**Definition 1** *Contextual centrality (CC) approximates the cascade payoff within a given number of time periods T as a function of the initial node accounting for individuals’ contribution to the cascade payoff*.

5 $${\rm{CC}}({\bf{A}},{\bf{P}},T,{\bf{y}}):=\mathop{\sum }\limits_{t=0}^{T}\,{({\bf{P}}\circ {\bf{A}})}^{t}{\bf{y}},$$

Heterogeneous diffusion rates across individuals are difficult to collect and estimate in real-world applications. Therefore, in the following analysis, we assume a homogeneous diffusion rate (*p*) across all edges. Hence, **P** ◦ **A** in Eq. (5) is reduced to *p***A**. Similar to diffusion centrality, contextual centrality is a random-walk-based centrality, where (*p***A**)^*t*^ measures the expected number of walks of length *t* between each pair of nodes and *T* is the maximum walk-length considered. Since *T* is the longest communication period, a larger *T* indicates a longer period for diffusion (e.g., a movie that stays in the market for a long period). In contrast, smaller *T* indicates a shorter diffusion period (e.g., a coupon that expires soon). On the one hand, when *pλ*_1_ is larger than 1, CC approaches infinity as *T* grows. On the other hand, when *pλ*_1_ < 1, CC is finite for *T* = ∞, which corresponds to a lack of virality, expressed in a fizzling out of the diffusion process with time. We can use the specific value of *pλ*_1_ to bound the maximum possible CC, given the norm of the score vector *y*. As presented in proposition 1 in the Supporting Information, the upper bound for CC grows with *pλ*_1_ and the norm of the score vector.

Let us further illustrate the relationship between CC and diffusion centrality, DC for short. In Banerjee *et al*.^12^, ${\rm{DC}}={\sum }_{t=1}^{T}{(p{\bf{A}})}^{t}$. To derive the following relationship between CC and DC, we add the score of reaching the first seeded individual into computing diffusion centrality. Hence, ${\rm{DC}}={\sum }_{t=0}^{T}{(p{\bf{A}})}^{t}$. Adding the first seeded individual into the scoring function produces the same ranking as the one used in Banerjee *et al*. We can represent **y** as, ${\bf{y}}=\sigma ({\bf{y}})\times {\bf{z}}+\bar{{\bf{y}}}\times 1$, where *σ*(**y**) and **z** are the standard deviation and the z-score normalization of **y**. Using the linearity of CC with respect to **y**, we can write

6 $${\rm{CC}}({\bf{A}},p,T,{\bf{y}})=\sigma ({\bf{y}})\cdot {\rm{CC}}({\bf{A}},p,T,{\bf{z}})+\bar{{\bf{y}}}\cdot \mathop{\underbrace{{\rm{CC}}({\bf{A}},p,T,1)}}\limits_{{\rm{DC}}}$$

Equation (6) shows the trade-off between the standard deviation *σ*(**y**) and the mean $\bar{{\bf{y}}}$ of the contribution vector in CC. When $\bar{{\bf{y}}}$ dominates over *σ*(**y**), network topology is more important in CC and it produces similar or opposite rankings to DC, depending on the sign of $\bar{{\bf{y}}}$. If the graph is Erdos-Renyi and *T* is small enough, then, on expectation, the term $\bar{{\bf{y}}}\cdot {\rm{DC}}$ dominates as the size of the network approaches infinity, as presented in Theorem 1 in the Supporting Information. However, when *σ*(**y**) dominates over $\bar{{\bf{y}}}$, CC and DC generate very different rankings.

The relevant node characteristics (**y)** provides the ex-ante estimation about one’s contribution. Whether to incorporate **y** is the main difference between our centrality and existing centrality measures. In the real-world data, the observation or estimation on **y** can be noisy, biased, or stochastic. Therefore, we discuss the robustness of contextual centrality in responses to perturbations in **y** in the Supporting Information.

We define the following terms, which we use throughout the paper:

- Spreadability (*pλ*_1_) captures the capability of the campaign to diffuse on the network depending on the diffusion probability (*p*) via a certain communication channel, and the largest eigenvalue (*λ*_1_) of the network.
- Standardized average contribution $(\frac{\bar{{\bf{y}}}}{\sigma ({\bf{y}})})$ is computed as the average of the contributions normalized by the standard deviation of the contributions. The sign of $\frac{\bar{{\bf{y}}}}{\sigma ({\bf{y}})}$ indicates whether the average contribution is positive or not. Moreover, the larger the magnitude of $\frac{\bar{{\bf{y}}}}{\sigma ({\bf{y}})}$, the more homogeneous the contributions are.
- Primary contribution $({{\bf{U}}}_{1}^{T}{\bf{y}})$ measures the joint distribution of the structural importance and nodal contributions. It captures whether people who dominate important positions have positive contributions or not.

## Properties of contextual centrality when *pλ*_1_ > 1 and *T* is large

Let us first provide the approximation of contextual centrality in this condition, which reveals one of the prominent advantages of contextual centrality. By the Perron-Frobenius Theorem, we have $|{\lambda }_{j}|\le {\lambda }_{1}$ for every *j*. Moreover, if we assume that the graph is non-periodic, then in fact $|{\lambda }_{j}| < {\lambda }_{1}$ for all *j* ≠ 1. Note that the typical random graph is not periodic, so this assumption is reasonable. Thus, when *pλ*_1_ > 1, the term (*pλ*_1_)^*t*^ grows exponentially faster than (*pλ*_*j*_)^*t*^ for *j* ≠ 1 so that the *j* = 1 term dominates for sufficiently large values of *T*, and we obtain the approximation for contextual centrality (CC_approx_):

7 $$\begin{array}{l}{\rm{CC}}=\mathop{\sum }\limits_{j=1}^{n}\,\mathop{\sum }\limits_{t=0}^{T}\,{(p{\lambda }_{j})}^{t}{{\bf{U}}}_{j}{{\bf{U}}}_{j}^{T}{\bf{y}}\approx {{\rm{CC}}}_{{\rm{approx}}}=(\mathop{\sum }\limits_{t=0}^{T}\,{(p{\lambda }_{1})}^{t}{{\bf{U}}}_{1}^{T}{\bf{y}}){{\bf{U}}}_{1}.\end{array}$$

This approximation reveals some desirable properties of contextual centrality. Crucially, CC_approx_ is simply a scalar multiple of the leading eigenvector when *pλ*_1_ > 1 and *T* is large. Therefore, the sign of ${{\bf{U}}}_{1}^{T}{\bf{y}}$ determines the direction of the relationship between CC_approx_ and eigenvector centrality. By Perron-Frobenius Theorem, all elements in this leading eigenvector are nonnegative. Thus, the approximated cascade payoff, Eq. (4), for seeding any individual is nonpositive if ${{\bf{U}}}_{1}^{T}{\bf{y}} < 0$, *pλ*_1_ > 1 and *T* is large. This shows that in this condition, the approximated cascade payoff is nonpositive for seeding any individual, so the campaigner should select a diffusion channel with a lower diffusion rate to take advantage of the local neighborhood with positive contributions. Equation (7) naturally suggests the following relationships between CC_approx_ and eigenvector centrality.

- If ${{\bf{U}}}_{1}^{T}{\bf{y}} > 0$, CC_approx_ and eigenvector centrality produce the same rankings.
- If ${{\bf{U}}}_{1}^{T}{\bf{y}} < 0$, CC_approx_ and eigenvector centrality produce the opposite rankings.

The approximation does not hold when ${{\bf{U}}}_{1}^{T}{\bf{y}}=0$, which is also unlikely to happen in practice. Hence, we disregard this case. Similarly, we relate contextual centrality to diffusion centrality (C_Diffusion_) and Katz centrality (C_Katz_),

8 $$\begin{array}{rcl}{{\rm{C}}}_{{\rm{Diffusion}}}\propto \mathop{\sum }\limits_{t=1}^{\infty }\,{(p{\lambda }_{1})}^{t}{{\bf{U}}}_{1}({{\bf{U}}}_{1}^{T}1) & = & \frac{{\sum }_{t=1}^{\infty }\,{(p{\lambda }_{1})}^{t}({{\bf{U}}}_{1}^{T}1)}{{\sum }_{t=0}^{T}\,{(p{\lambda }_{1})}^{t}{{\bf{U}}}_{1}^{T}{\bf{y}}}{{\rm{CC}}}_{{\rm{approx}}},\\ {{\rm{C}}}_{{\rm{Katz}}}\propto \mathop{\sum }\limits_{t=0}^{\infty }\,{(\alpha {\lambda }_{1})}^{t}{{\bf{U}}}_{1}({{\bf{U}}}_{1}^{T}1) & = & =\frac{{\sum }_{t=0}^{\infty }\,{(\alpha {\lambda }_{1})}^{t}({{\bf{U}}}_{1}^{T}1)}{{\sum }_{t=0}^{T}\,{(p{\lambda }_{1})}^{t}{{\bf{U}}}_{1}^{T}{\bf{y}}}{{\rm{CC}}}_{{\rm{approx}}},\end{array}$$

where *α* is the decay factor in Katz centrality. Similar to Eq. (7), all terms on the right-hand-side of Eq. (8) are positive except for ${{\bf{U}}}_{1}^{T}{\bf{y}}$, which similarly determines the direction of the relationship.

## Results

### Predictive power of contextual centrality

We study two real-world empirical settings, adopting microfinance in 43 Indian villages^12^ and adopting weather insurance in 47 Chinese villages^17^. In each setting, there is a set of first-informed households in each village who went on to spread the information. We compare contextual centrality with diffusion centrality and other widely-adopted reachability-based centrality measures – degree, eigenvector, and Katz centrality. We compute degree centrality by taking the degree of each node, normalized by *N* − 1. We compute eigenvector centrality by taking the leading eigenvector **U**_1_ with unit length and nonnegative entries. We compute Katz centrality as ${\sum }_{t=0}^{\infty }{(\alpha {\bf{A}})}^{t}1$, setting *α*, which should be strictly less than ${\lambda }_{1}^{-1}$, to $0.9\cdot {\lambda }_{1}^{-1}$. We compute diffusion centrality as ${\sum }_{t=1}^{T}{(p{\bf{A}})}^{t}1$. For both diffusion and contextual centrality, we set *T* = 16, except for the microfinance in Indian villages setting, where we set *T* the same as Banerjee *et al*.^12^. We evaluate the adoption outcome of all other households in the village, which are not first-informed. We use the adoption likelihood for the contribution vector **y** in computing contextual centrality, which is predicted using a model based on the adoption decisions of the first-informed households. In the empirical analysis of both settings, we build models to predict the adoption likelihood for each individual to use as **y** in computing contextual centrality. For each setting, we use the data provided in Banerjee *et al*.^12^ and Cai *et al*.^17^, respectively, as inputs to a feed-forward neural network trained to predict the adoption likelihood based on the adoption decisions of first-informed individuals. Hyperparameters, including hidden layers, activation function, and regularization, were tuned using grid search with 10-fold cross-validation. For the microfinance in Indian villages, the covariates include village size, quality of access to electricity, quality of latrines, number of beds, number of rooms, the number of beds per capita, and the number of rooms per capita. For the weather insurance in Chinese villages setting, 39 of the provided characteristics are selected as inputs by choosing those for which all households had data after removing households with many missing characteristics. Similar to Banerjee *et al*.^12^, we evaluate the *R*^2^ of a linear regression model for both settings. The independent variables include the average centrality of first-informed households and the village size, a control variable. The dependent variable is the fraction of non-first-informed households in a village which adopted.

**Figure 1:**
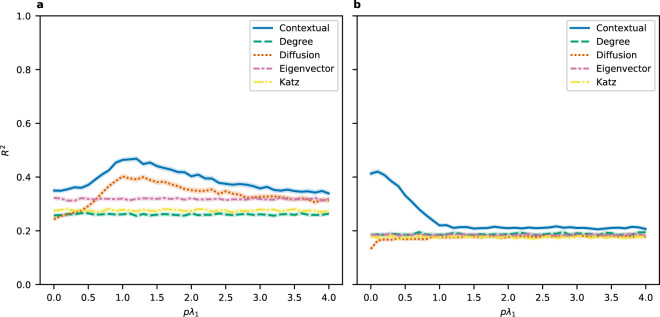
Predictive power of contextual centrality. We show how the average centrality of first-informed individuals predicts the eventual adoption rate of non-first-informed individuals in (**a**) microfinance and (**b**) weather insurance. The y-axis shows the 95% confidence interval of *R*^2^ computed from 1000 bootstrap samples from ordinary least squares regressions controlling for village size. The x-axis shows varying values for *pλ*_1_, which influences only diffusion centrality and contextual centrality.

In Fig. 1, we show how the *R*^2^ for various measures of centrality varies with *pλ*_1_, in which the choice of *p* influences the two centrality measures that account for the diffusion process - diffusion centrality and contextual centrality. We see that the contextual centrality outperforms all other standard centrality measures, which indicates that marketing campaigners or social planners will benefit from using contextual centrality as the seeding strategy to maximize participation. This result also highlights that utilizing ex-ante information about customers’ likelihood of adoption helps to design better targeting strategies. Similar results without control variables and with more control variables are presented in the Supporting Information as a robustness check.

### Performance of contextual centrality relative to other centrality measures on random networks

To better understand CC’s performance with respect to different parameters (*pλ*_1_, $\frac{\bar{{\bf{y}}}}{\sigma ({\bf{y}})}$), we next perform simulations on randomly generated synthetic networks and contribution vectors (**y**). For the synthetic setting, we generate a new random graph for each simulation, according to Barabasi-Albert, Erdos-Renyi, and Watts-Strogatz models. The size of *n* of each graph varies between 30 and 300. For Barabasi-Albert, *m* varied between 1 and *n*. For Erdos-Renyi, *p* varies between 0 and 1. For Watts-Strogatz, *k* varies between ln *n* and *n*, and *p* varies between 0 and 1. Individual contributions **y** are sampled from a normal distribution with unit standard deviation. Note that the scale of **y** does not change the rankings of contextual centrality. Simulations of the diffusion process in each setting follow the independent cascade model^13^. For each centrality, the highest-ranked node is set to be the initial seed. We compute cascade payoff by summing up the individual contributions of all the nodes reached in the cascade. For each parameter tested in different settings, we run 100 simulations. To compare the performance of contextual centrality against the other centrality measures, we use “relative change” (calculated as $\frac{a-b}{\max (|\,a\,|,|\,b\,|)}$, where *a* is a given centrality’s average payoff and *b* is the maximum average payoff of the other centrality measures). We chose “relative change” for comparison since it gives a sense of when the payoffs are different from the optimal centrality while keeping the magnitudes of the payoffs in perspective. This measure has some desirable properties. First, its value is necessarily between −2 and 2, so our scale for comparison is consistent across scenarios. Second, its magnitude does not exceed one unless *a* and *b* differ in sign, so we can tell if a centrality gets a positive average payoff while the rest do not.

**Figure 2:**
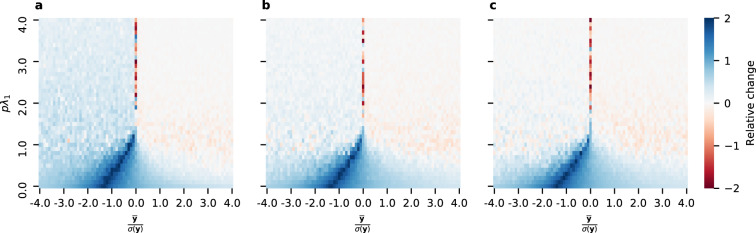
Performance of contextual centrality relative to other centrality measures on random networks. Each plot shows the relative change, computed as $\frac{a-b}{\max (|a|,|b|)}$ where *a* is CC’s average payoff and *b* is the maximum average payoff of the other centrality measures, for varying values of $\frac{\bar{{\bf{y}}}}{\sigma ({\bf{y}})}$ and *pλ*_1_. The plots correspond to the results on random networks generated according to the (**a**) Barabasi-Albert, (**b**) Erdos-Renyi, and (**c**) Watts-Strogatz models.

Figure 2 displays the relative change between CC’s average payoff and the maximum average payoff of the other centrality measures aggregated over 100 runs of simulations for varying values of $\frac{\bar{{\bf{y}}}}{\sigma ({\bf{y}})}$ and *pλ*_1_ on three different types of simulated graphs. We can see that CC performs well when $\bar{{\bf{y}}} < 0$, *pλ*_1_ < 1, and $\frac{\bar{{\bf{y}}}}{\sigma ({\bf{y}})}$ is small in magnitude. We will now discuss each of these cases in more detail.

When $\bar{{\bf{y}}} < 0$, maximizing the reach of the cascade is not ideal because that will result in a cascade payoff, which more closely reflects $\bar{{\bf{y}}}$. CC differs from the other centrality measures in that it does not try to maximize the reach of the cascade. Note the dark blue diagonal band present in all plots in Fig. 2. Since the magnitude of the relative change exceeds one only when the values being compared have opposite signs, this region shows that there are many settings where the standardized average contribution is negative, nevertheless CC achieves a positive average payoff while the other centrality measures do not.

When *pλ*_1_ is small, it is essential to seed an individual whose local neighborhood has higher individual contributions since there is not much risk of diffusing to individuals with lower individual contributions As an extreme case, consider *pλ*_1_ = 0. In this case, the diffusion rate is 0, so seeding an individual with a high individual payoff makes much more sense than seeding an individual with high topological importance. This highlights CC’s advantage in discriminating the local neighborhoods with positive payoffs from those with negative payoffs while the other centrality measures cannot.

When $\frac{\bar{{\bf{y}}}}{\sigma ({\bf{y}})}$ is small in magnitude, CC takes advantage of the greater relative variations between contributions. As $\frac{\bar{{\bf{y}}}}{\sigma ({\bf{y}})}\to +\,\infty$, Eq. (6) tells us that CC will seed similar to DC, which explains why CC loses some of its advantage. However, as $\frac{\bar{{\bf{y}}}}{\sigma ({\bf{y}})}\to -\,\infty$, Eq. (6) tells us that CC will seed opposite to DC, which explains why CC maintains an advantage.

**Figure 3:**
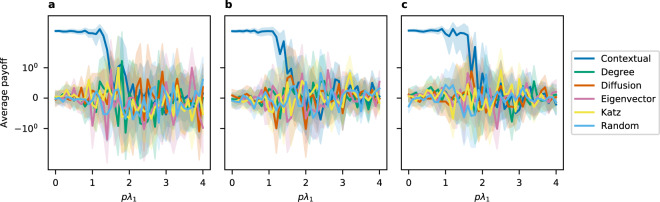
Average payoffs when standardized average contribution is 0. Here we show the average payoff with 95% confidence interval when seeding with different methods on (**a**) Barabasi-Albert, (**b**) Erdos-Renyi, and (**c**) Watts-Strogatz models.

**Figure 4:**
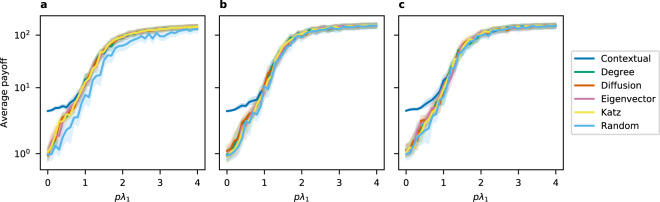
Average payoffs when standardized average contribution is 1. Here we show the average payoff with 95% confidence interval when seeding with different methods on (**a**) Barabasi-Albert, (**b**) Erdos-Renyi, and (**c**) Watts-Strogatz models.

We now discuss the regions where CC does not seem to offer an advantage. Note that parameters for which CC’s average payoff is lower than that of some other centrality often neighbor similar parameters for which CC’s average payoff is the same, or sometimes higher, than those of the other centrality measures. This suggests that CC is performing comparably, which is what we expect as *pλ*_1_ increases since the initial seed matters less as the diffusion process reaches more individuals. In Figs. 3 and 4, we show the average payoffs of different seeding methods with 95% confidence interval when the standardized average contribution is 0 and 1, respectively, on (a) Barabasi-Albert, (b) Erdos-Renyi, and (c) Watts-Strogatz models. Note that when *pλ*_1_ is small, CC dominates the other seeding methods. As *pλ*_1_ increases, CC’s performance is on par with other centrality measures, as can be seen from the highly overlapping confidence intervals. This pattern holds for other values of the standardized average contribution. Similar figures to Figs. 3 and 4 for other values of the standardized average contribution can be found in the Supporting Information.

### Performance of contextual centrality relative to other centrality measures on real-world networks

**Figure 5:**
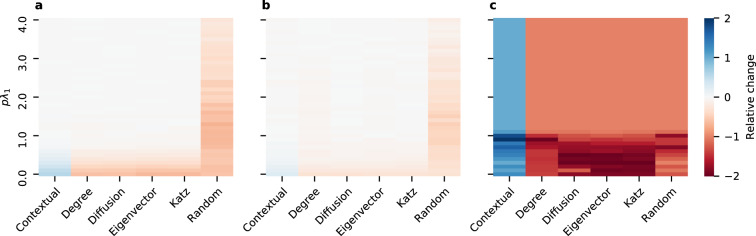
Performance of contextual centrality relative to other centrality measures on real-world networks, including (**a**) microfinance, (**b**) weather insurance, and (**c**) political campaign. Each plot shows the relative change for varying values of *pλ*_1_. We compare contextual centrality with degree centrality, diffusion centrality, eigenvector centrality, Katz centrality, and random seeding.

Next, we analyze the performance of contextual centrality in achieving the cascade payoff, as defined in Eq. (3), using simulations on three real-world settings, namely adoption of microfinance, adoption of the weather insurance, and political voting campaign, as shown in Fig. 5. For the political campaign experiment in Turkey, we use individual home and work locations to build a network and regional voting data on sampling voting outcomes to use as **y**. Individuals belonging to the same home neighborhood are connected according to the Watts-Strogatz model with a maximum of 10 neighbors. Same for the work neighborhoods. These two networks are superimposed to form the final network. Since we do not know the political voting preferences on an individual level, individual voting outcomes are sampled to match voting data on a regional level. Specifically, we let the actual fraction of the population that voted for the AK Party in an individual’s home neighborhood be the probability that individual votes for the AK Party. We let *y*_*i*_ = +1 represent a vote for AK party and *y*_*i*_ = −1 represent a vote for any other party. We sample a new set of voting outcomes from the regional voting distributions for each diffusion simulation.

To compare the performance of contextual centrality against the maximum of centrality measures for each condition, we use “relative change” as before. We observe the network structure (**A**) and adoption decisions in the campaign for microfinance and weather insurance. In the campaign for political votes, we generate the network structure and the contribution vector from the empirical distributions. We vary the diffusion rate of *p* in the independent cascade model to examine how it influences the performances of different centrality measures. We see that in (a) campaign for microfinance and (b) campaign for weather insurance, CC outperforms the other centrality measures when *pλ*_1_ is small. While in (c) campaign for political votes, CC outperforms the other centrality measures for all *pλ*_1_. The standardized average contributions of (a), (b), and (c) are 2.29, 5.27, and −2.22, respectively. This result is consistent with the results presented in Fig. 2. It shows that contextual centrality can greatly outperform other centrality measures when the standardized average contribution is negative for a wide range of *pλ*_1_. When standardized average contribution is positive, contextual centrality outperforms other centrality measures when the spreadability is small and achieves comparable results with other centrality measures as the spreadability further increases.

### Approximation of contextual centrality and the importance of primary contribution

**Figure 6:**
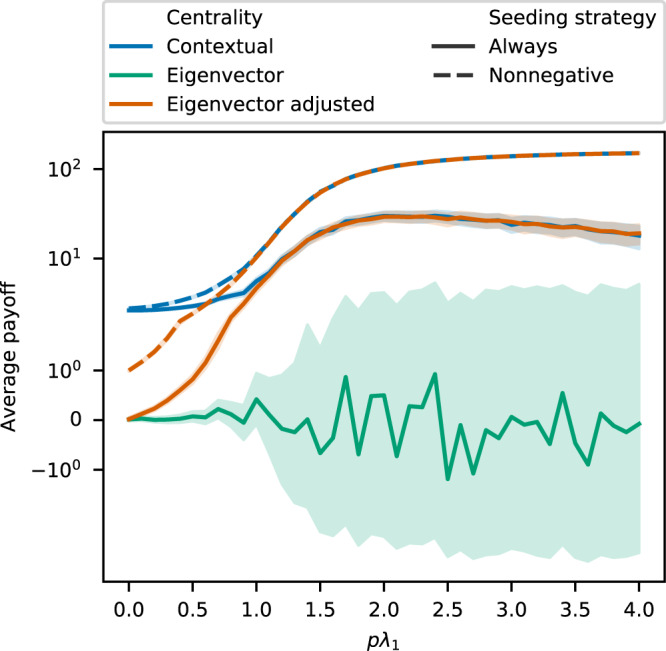
Average cascade payoff for variations of contextual centrality and eigenvector centrality. The x-axis is *pλ*_1_, and the y-axis is the average payoff, with the shaded region as the 95% confidence intervals. For “eigenvector adjusted” centrality, we multiply eigenvector centrality with the primary contribution ${{\bf{U}}}_{1}^{T}{\bf{y}}$. For “Nonnegative”, we only seed if the maximum of the centrality measure is nonnegative, otherwise it is named “Always”.

A negative contextual centrality score indicates that seeding with the particular node will generate a negative payoff. Therefore, we design a seeding strategy in which we seed only if the maximum of contextual centrality is nonnegative. As shown by the blue dashed and solid lines in Fig. 6, the new seeding strategy, “Nonnegative”, performs better than always seeding the top-ranked individual. Building upon Eq. (7), we introduce a variation of eigenvector centrality, “Eigenvector adjusted”, as the product of eigenvector centrality and the primary contribution (${{\bf{U}}}_{1}^{T}{\bf{y}}$). This variation of eigenvector centrality performs on par with contextual centrality as *pλ*_1_ grows large as expected according to Eq. (7). “Eigenvector adjusted” greatly outperforms eigenvector centrality. Another variation of eigenvector centrality is to adjust eigenvector centrality by $\bar{{\bf{y}}}$. Note that the sign of ${{\bf{U}}}_{1}^{T}{\bf{y}}$ does not always equal $\bar{{\bf{y}}}$. When the signs differ, seeding only when ${{\bf{U}}}_{1}^{T}{\bf{y}}$ is positive produces a higher cascade payoff when *pλ*_1_ is not too large. However, as *pλ*_1_ further increases and the diffusion saturates most of the network, the sign of $\bar{{\bf{y}}}$ predicts that of the cascade payoff. However, larger *pλ*_1_ is not as interesting as smaller ones, which happens more frequently in real life. We present average cascade payoff comparing the two strategies when $\bar{{\bf{y}}}({{\bf{U}}}_{1}^{T}{\bf{y}}) < 0$ in the Supplementary Information.

Comparing the strategies in Fig. 6, the new strategy of accounting for the sign of the centrality measures improves the average payoffs by an order of magnitude. This pattern also highlights the importance of the primary contribution to campaign strategies. We present figures for the analogous variations of the other centrality measures in the Supporting Information.

### Homophily and the maximum of contextual centrality

**Figure 7:**
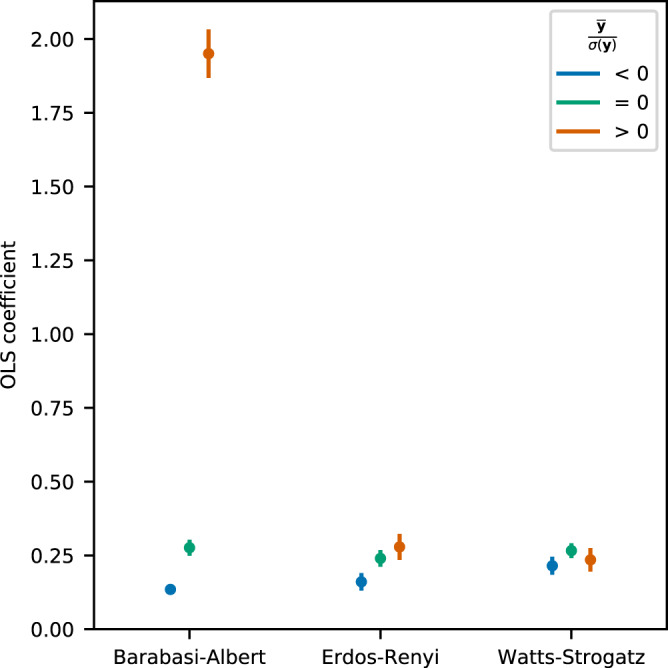
Homophily and maximum of contextual centrality when *pλ*_1_ < 1. We regress the maximum of contextual centrality on homophily after controlling for $\frac{\bar{{\bf{y}}}}{\sigma ({\bf{y}})}$ and *pλ*_1_. The y-axis is the OLS coefficients of homophily (with the vertical line as the 95% confidence interval) and the x-axis corresponds to three types of networks. We perform the analysis separately for $\frac{\bar{{\bf{y}}}}{\sigma ({\bf{y}})}$ being larger than, smaller than and equals to zero.

Homophily is a long-standing phenomenon in social networks that describes the tendency of individuals with similar characteristics to associate with one another^18^. The strength of homophily is measured by the difference in the contributions of the neighbors, ${\sum }_{i,j}^{N}{A}_{ij}{({y}_{i}-{y}_{j})}^{2}$. We analyze the relationship between the strength of homophily and the approximated cascade payoff by seeding the highest-ranked node in contextual centrality in Fig. 7. After controlling for $\frac{\bar{{\bf{y}}}}{\sigma ({\bf{y}})}$ and *pλ*_1_, we regress the maximum of the contextual centrality on the strength of homophily of the network separately for three conditions of $\frac{\bar{{\bf{y}}}}{\sigma ({\bf{y}})}$. When the spreadability of contextual centrality is small, stronger homophily tends to correlate with a large approximated cascade payoff across all graph types. This result shows that stronger homophily of the network predicts higher approximated cascade payoff with small spreadability. When the network is Barabasi-Albert and $\frac{\bar{{\bf{y}}}}{\sigma ({\bf{y}})} > 0$, the relationship is the strongest. As the spreadability further increases, the correlation between contextual centrality and homophily drops dramatically, and thereby we exclude the scenarios when *pλ*_1_ > 1.

## Discussion

Contextual centrality sheds light on the understanding of node importance in networks by emphasizing node characteristics relevant to the objective of the diffusion other than the structural topology, which is vital for a wide range of applications, such as marketing or political campaigns on social networks. Notably, nodal contributions to the objective, the diffusion probability, and network topology jointly produce an effective campaign strategy. It should now be clear with the thorough simulations and empirical analysis in this study that exposing a large portion of the population in the diffusion is not always desirable.

- When the spreadability is small, contextual centrality effectively ranks the nodes whose local neighborhoods generate larger cascade payoffs the highest.
- When the spreadability is large, the primary contribution tends to predict the sign of the approximated cascade payoff.

Meanwhile, for a given contribution vector (**y**), the policy-maker can influence the diffusion rate to take advantage of local diffusion and locate nodes whose local neighborhood generates large cascade payoff. In practice, the policy-maker can first estimate the contribution vector (**y**), and then calculate the maximum of contextual centrality for a range of *pλ*_1_, which approximates the cascade payoff. Finally, the policy-maker can compute the optimal corresponding *p* given the leading eigenvector (*λ*_1_).

When the primary contribution is negative, the campaigner might need to reduce the spreadability of the campaign to take advantage of the individuals whose local neighborhoods generate positive approximated cascade payoff in aggregation. To reduce the spreadability of the campaign, the campaigner can resort to campaign channels with lower diffusion probability and less viral features, such as direct mail.

As the standardized average contribution increases, the contribution vector becomes comparatively more homogeneous and comparatively less important than the network structure. Therefore, when the average contribution is positive, seeding with contextual centrality becomes similar to seeding with diffusion centrality.

Moreover, contextual centrality emphasizes the importance of incorporating node characteristics that are exogenous to the network structure and the dynamic process. More broadly, contextual centrality provides a generic framework for future studies to analyze the joint effect of network structure, nodal characteristics, and the dynamic process. Other than applications on social networks, contextual centrality can be applied to analyzing a wide range of networks, such as the biology networks (e.g., rank the importance of genes by using the size of their evolutionary family as the contribution vector^19^), the financial networks (e.g., rank the role of institutions in risk propagation in financial crisis with their likelihoods of failure as the contribution vector^3^), and the transportation networks (e.g., rank the importance of airports with the passengers flown per year as the contribution vector^20^).

## Methods

In this study, we compare contextual centrality with diffusion centrality and other widely adopted reachability-based centrality measures - degree, eigenvector, and Katz centrality. We compute degree centrality by taking the degree of each node, normalized by *N* − 1. We compute eigenvector centrality by taking the leading eigenvector **U**_1_ with unit length and nonnegative entries. We compute Katz centrality as ${\sum }_{t=0}^{\infty }{(\alpha {\bf{A}})}^{t}1$, setting *α*, which should be strictly less than ${\lambda }_{1}^{-1}$, to $0.9\cdot {\lambda }_{1}^{-1}$. We compute diffusion centrality as ${\sum }_{t=1}^{T}{(p{\bf{A}})}^{t}1$. For both diffusion and contextual centrality, we set *T* = 16, except for the microfinance in Indian villages setting, where we set *T* as done by Banerjee *et al*.^12^.

Simulations of the diffusion process in each setting follow the independent cascade model^13^. For each centrality, the highest-ranked node is set to be the initial seed. We compute cascade payoff by summing up the individual contributions of all the nodes reached in the cascade. For each parameter tested in different settings, we run 100 simulations.

In the empirical analysis of microfinance in Indian villages and weather insurance in Chinese villages, we build models to predict the adoption likelihood to use as **y** in computing contextual centrality. For each setting, we use the data provided in Banerjee *et al*.^12^ and Cai *et al*.^17^, respectively, as inputs to a feed-forward neural network trained to predict the adoption likelihood based on the adoption decisions of first-informed individuals. For the microfinance in Indian villages, the covariates include village size, quality of access to electricity, quality of latrines, number of beds, number of rooms, the number of beds per capita, and the number of rooms per capita. For the weather insurance in Chinese villages setting, 39 of the provided characteristics are selected as inputs by choosing those for which all households had data after removing households with many missing characteristics.

For the political campaign experiment in Turkey, we use individual home and work locations to build a network and regional voting data on sampling voting outcomes to use as **y**. Individuals belonging to the same home neighborhood are connected according to the Watts-Strogatz model with a maximum of 10 neighbors. Same for the work neighborhoods. These two networks are superimposed to form the final network. Since we do not know the political voting preferences on an individual level, individual voting outcomes are sampled to match voting data on a regional level. Specifically, we let the actual fraction of the population that voted for the AK Party in an individual’s home neighborhood be the probability that an individual votes for the AK Party. We let *y*_*i*_ = +1 represent a vote for AK party and *y*_*i*_ = −1 represent a vote for any other party. We sample a new set of voting outcomes from the regional voting distributions for each diffusion simulation.

For the synthetic setting, we generate a new random graph for each simulation, according to Barabasi-Albert, Erdos-Renyi, and Watts-Strogatz models. The size *n* of each graph varies between 30 and 300. For Barabasi-Albert, *m* varied between 1 and *n*. For Erdos-Renyi, *p* varies between 0 and 1. For Watts-Strogatz, *k* varies between ln *n* and *n*, and *p* varies between 0 and 1. Individual contributions **y** are sampled from a normal distribution with unit standard deviation. Note that the scale of **y** does not change the rankings of contextual centrality.

## Supplementary information

Supplementary Information.
