# Supplementary material for: Contextual centrality: going beyond network structure
Source: Sci Rep. 2020 Jun 10;10:9401. doi: 10.1038/s41598-020-62857-4 (PMC7286920; doi:10.1038/s41598-020-62857-4)
Supplement: Supplementary file 1 — Supplementary Information. [file 41598_2020_62857_MOESM1_ESM.pdf]

# Supporting Information: Contextual centrality: going beyond network structure

Yan Leng [1†], Yehonatan Yella[2], Rodrigo Ruiz[1], Alex Pentland[1]

[1]Massachusetts Institute of Technology, Cambridge, MA, USA
[2]Albert Einstein College of Medicine, New York, USA
[†] To whom correspondence should be addressed; E-mail: yleng@mit.edu

## Contents

**1 Properties of contextual centrality** **2**

1.1 Bounds and distribution of contextual centrality in terms of spreadability . . . . . . . . . 2

1.2 Robustness of contextual centrality in response to perturbations in $\mathbf{y}$ . . . . . . . . . . . 3

1.2.1 Sensitivity Analysis . . . . . . . . . . . . . . . . . . . . . . . . . . . . . 3

1.2.2 Contextual centrality as a random variable . . . . . . . . . . . . . . . . . . . . . 4

1.3 Theoretical results of contextual centrality for Erdos-Renyi networks . . . . . . . . . . . 4

1.4 The relationship between contextual centrality and other centrality measures . . . . . . . 11

1.5 Relationship between approximated cascade payoff and cascade payoff . . . . . . . . . 12

1.6 Game-theoretic interpretation of contextual centrality with local interactions . . . . . . . 13

1.7 Differences between contextual centrality and centrality measures developed on weighted networks . . . . . . . . . . . . . . . . . . . . . . . . . . . . . . . . . . . . 14

**2 Supplementary figures for empirical analysis** **16**

2.1 Predictive power of contextual centrality in eventual adoptions . . . . . . . . . . . . . . 16

2.2 Performance relative to other centrality measures on random networks . . . . . . . . . . 18

2.3 Average approximated cascade payoff for contextual centrality and the variations of other centrality measures . . . . . . . . . . . . . . . . . . . . . . . . . . . . . . . 22

2.4 Comparison of seeding strategies when $\overline{\mathbf{y}}(\mathbf{U}_1^T\mathbf{y}) < 0$ . . . . . . . . . . . . . . . . . . 24

# 1 Properties of contextual centrality

## 1.1 Bounds and distribution of contextual centrality in terms of spreadability

In this section, we present the upper bound for maximum possible contextual centrality. When $p\lambda_1$ is larger than 1, CC approaches infinity as $T$ grows. On the other hand, when $p\lambda_1 < 1$, CC is finite for $T = \infty$, which can be understood as a lack of virality, expressed in a fizzling out of the diffusion process with time. We can use the value of $p\lambda_1$ to bound the maximum possible CC given the norm of the score vector $\mathbf{y}$.

**Proposition 1**

$$max(CC(\mathbf{A}, p, T, \mathbf{y})) \leq ||CC(\mathbf{A}, p, T, \mathbf{y})||$$
$$\leq \frac{1 - (p\lambda_1)^{T+1}}{1 - p\lambda_1} ||\mathbf{y}||$$

*If, in addition,* $p\lambda_1 < 1$, *then this is further bounded by* $\frac{1}{1-p\lambda_1}||\mathbf{y}||$.

**Proof 1** *The first inequality,* $max(CC(\mathbf{A}, p, T, \mathbf{y})) \leq ||CC(\mathbf{A}, p, T, \mathbf{y})||$, *is clear.*

*Next we use the matrix norm* $||\mathbf{A}|| := sup\{||\mathbf{A}x||/||x|| : x \neq 0\}$, *which by definition satisfies* $||\mathbf{A}x|| \leq ||\mathbf{A}|| \cdot ||x||$ *for all* $x$, *and which coincides with spectral radius* $\rho(\mathbf{A})$ *for symmetric matrices. Since, for us,* $\rho(\mathbf{A}) = \lambda_1$, *we have*

$$||CC(\mathbf{A}, p, T, \mathbf{y})|| = ||(\sum_{t=0}^{T}(p\mathbf{A})^t)\mathbf{y}|| \leq ||(\sum_{t=0}^{T}(p\mathbf{A})^t)|| \cdot ||\mathbf{y}||$$
$$\leq \sum_{t=0}^{T} ||(p\mathbf{A})^t|| \cdot ||\mathbf{y}|| = \sum_{t=0}^{T}(p\lambda_1)^t \cdot ||\mathbf{y}|| \leq \frac{1 - (p\lambda_1)^{T+1}}{1 - p\lambda_1}||\mathbf{y}||$$

*which, if* $p\lambda_1 < 1$, *can be further bounded by* $\frac{1}{1-p\lambda_1}||\mathbf{y}||$

While the above result bounds contextual centrality from above, the actual value of CC is highly variable, depending on the structure of the graph and the distribution of the score vector among its nodes. For a discussion of expected CC among random networks, see the Erdos-Reyni section below. Next, we discuss the behavior of contextual centrality when $\mathbf{y}$ is variable.

## 1.2 Robustness of contextual centrality in response to perturbations in $\mathbf{y}$

As discussed in the main body of the paper, in real-world data, node characteristics can be noisy, stochastic, and biased. Therefore, it is essential to analyze the robustness of contextual centrality in response to small perturbations in $\mathbf{y}$. We first perform a sensitivity analysis, studying bounds on the error in contextual centrality in terms of noise in $\mathbf{y}$, and then study contextual centrality as a random variable assuming a multivariate normal model of $\mathbf{y}$.

### 1.2.1 Sensitivity Analysis

We let the observed (or estimated) score vector be $\hat{\mathbf{y}}$ and let $\mathbf{y}$ be the true score vector. The errors in the score vector are given by the vector $\Delta\mathbf{y} := \mathbf{y} - \hat{\mathbf{y}}$ and similarly $\Delta\text{CC} := \text{CC}(\mathbf{A}, p, T, \hat{\mathbf{y}}) - \text{CC}(\mathbf{A}, p, T, \mathbf{y})$ is the error between the CC computed from observed and actual data.

We have the following bound on $||\Delta\text{CC}||$, which follows directly from Proposition 1 and the fact that CC is linear with respect to the score vector $\mathbf{y}$.

**Corollary 1**

$$||\Delta CC|| \leq \frac{1 - (p\lambda_1)^{T+1}}{1 - p\lambda_1} ||\Delta\mathbf{y}||$$

*If, in addition,* $p\lambda_1 < 1$, *then this is further bounded by* $\frac{1}{1-p\lambda_1}||\Delta\mathbf{y}||$.

This shows that when $p\lambda_1 < 1$, then as long as the error in $\mathbf{y}$ is sufficiently small, the error in CC will be small as well. However, the larger $p\lambda_1$ is, the more a small error in $\mathbf{y}$ can become amplified as an error in CC.

Next we focus on the case that $p\lambda_1 > 1$. In this case, we have shown in the main body of the paper that for large T, contextual centrality is well-approximated by $(\mathbf{U}_1^T\mathbf{y})\mathbf{U}_1$, where $\mathbf{U}_1$ is the eigenvector with the largest eigenvalue. Thus, in this case, the primary contribution $\mathbf{U}_1^T\mathbf{y}$ is an essential quantity whose sign roughly determines the relative ranking of contextual centrality. Hence, we analyze its sensitivity to noise in $\mathbf{y}$. The error in primary contribution is simply $\mathbf{U}_1^T\Delta\mathbf{y}$, whose magnitude is bounded by $||\Delta\mathbf{y}||$.

Thus if $\Delta\mathbf{y}$ is small enough so that $||\Delta\mathbf{y}|| < \mathbf{U}_1^T\hat{\mathbf{y}}$, this perturbation will not affect the sign of the primary contribution, so the relative ranking in CC will tend to stay fixed. Otherwise, the relative ranking is at risk of flipping.

### 1.2.2 Contextual centrality as a random variable

Next, to study the impact of stochasticity in $\mathbf{y}$, we suppose that $\mathbf{y}$ is a multivariate random variable with mean vector $\hat{\mathbf{y}}$ and covariance matrix $\Sigma$. Let $\mathbf{B} := \sum_{t=0}^{T}(p\mathbf{A})^t$. Since $\text{CC}(\mathbf{A}, p, T, \mathbf{y}) = \mathbf{B}\cdot\mathbf{y}$ is a linear transformation of the multivariate normal variable $\mathbf{y}$, it is also a multivariate normal variable, with mean $\mathbf{B}\hat{\mathbf{y}} = \text{CC}(\mathbf{A}, p, T, \hat{\mathbf{y}})$ and covariance matrix $\mathbf{B\Sigma B}$.

To simplify, consider the case that $\mathbf{\Sigma} = \sigma^2\mathbf{I}$, that is, the $y_i$ are uncorrelated and have the same standard deviation $\sigma$. Then the covariance matrix of $\text{CC}(\mathbf{A}, p, T, \mathbf{y})$ is $\sigma^2\mathbf{B}^2$.

That is, we have

$$\text{Cov}(\text{CC}(\mathbf{A}, p, T, \mathbf{y})_i, \text{CC}(\mathbf{A}, p, T, \mathbf{y})_j) = \sigma^2(\mathbf{B}e_i)\cdot(\mathbf{B}e_j),$$

where $e_i$ are the standard basis vectors.

In particular, the coefficients of CC may be positively correlated even when those of $\mathbf{y}$ are uncorrelated, and their standard deviations are given by

$$\sigma(\text{CC}(\mathbf{A}, p, T, \mathbf{y})_i) = \sigma||\mathbf{B}e_i||$$

Note that, by definition of $\mathbf{B}$, $\mathbf{B}e_i = \text{CC}(\mathbf{A}, p, T, e_i)$, whose $j$th coefficients represents the expected number of times node $i$ is reached by the diffusion process, if seeded at node $j$.

By Proposition 1, we have the bound $\sigma(\text{CC}(\mathbf{A}, p, T, \mathbf{y})_i) = \sigma||\mathbf{B}e_i|| \leq \frac{\sigma}{1-p\lambda_1}$ if $p\lambda_1 < 1$.

## 1.3 Theoretical results of contextual centrality for Erdos-Renyi networks

In the case that $\mathbf{A}$ corresponds to an Erdos-Renyi graph $G(n, q)$, we have further theoretical results, in line with the results of Banerjee et al.[1] on diffusion centrality. As is standard for Erdos-Renyi graphs,

we assume each edge has independent probability $q$ of being present in the graph, where $q$ is a function of $n$, the number of nodes. Assume that $qn$ grows such that $log(n) \leq qn \leq \sqrt{n}$. We also assume that $T$ and $p$ are functions of $n$, and let $\mathbf{y}$ denote the vector (depending on $n$) consisting of $y_1, \ldots, y_n$ for some infinite sequence $\{y_i\}$. We suppress all dependency on $n$ for ease of notation. We further assume that the mean $\overline{\mathbf{y}}$ has a limit $\overline{\mathbf{y}}$ as $n$ approaches infinity, which is reasonable by the law of large numbers if the $y_i$ are sampled from a random variable. With this background, we study the expected behavior of $E(\text{CC}(\mathbf{A}, p, T, \mathbf{y}))$.

Given two functions $f(n), g(n)$, we will say that $f$ approaches $g$ as $n$ approaches infinity, if $lim_{n\to\infty} \frac{f(n)}{g(n)} = 1$. Then we have the following result.

**Theorem 1** *Suppose $T = o(qn)$ and $\log(n) \leq qn \leq \sqrt{n}$. Then we can decompose $E(CC(\mathbf{A}, p, T, \mathbf{y})_i) = \overline{\mathbf{y}}E_1 + y_iE_2$, where $E_1$ and $E_2$ are functions of $n, p, q, T$ but do not depend on $\mathbf{y}$ or $i$, such that*

*a)$E_1$ approaches $\frac{1-(npq)^{T+1}}{1-(npq)}$.*

*b)$E_2 = o(E_1)$.*

*c)If $\overline{\mathbf{y}} \neq 0$, then $E(CC(\mathbf{A}, p, T, \mathbf{y}))$ approaches $\overline{\mathbf{y}}E(DC(\mathbf{A}, p, T, \mathbf{y}))$, where DC is diffusion centrality.*

In other words, if $\overline{\mathbf{y}} \neq 0$, then the term $E_1$ dominates, so the expected CC is uniform all nodes (in the limit as $n$ approaches infinity). Moreover, $\overline{\mathbf{y}}$ measures the magnitude of the diffusion as compared to DC, and the sign of $\overline{\mathbf{y}}$ determines the expected sign of CC. In contrast, if $\overline{\mathbf{y}} = 0$, then CC equals $E_2$ so, on expectation, CC correlates perfectly with $\mathbf{y}$ itself. We note that in practice it is not likely for $\overline{\mathbf{y}}$ to equal 0. However, if $\overline{\mathbf{y}}$ is close to 0 and $n$ is not too large, then the term $E_2$ could still be significant, indicating that the expected CC will be correlated with the nodal evaluation vector $\mathbf{y}$.

This result can also be related to the tradeoff in Eq. (6). As implied by the Theorem, as long as $\bar{y} \neq 0$, then expected CC approaches $\bar{Y}E_1$, which in turn approaches $\bar{y}E(\text{DC})$ as $n$ approaches infinity. Thus the second term of the tradeoff in Eq. (6) dominates, on expectation.

We also note that careful analysis will show that $E_2 > 0$, but that is beyond the scope of the present paper.

**Theorem 2** *If $p\lambda_1 \geq (1+\epsilon)$ for some $\epsilon > 0$, then $T = \frac{log(n)}{log(npq)}$ is a threshold for viral spread if $\overline{\mathbf{y}} \neq 0$, in the sense that*

*a)If $T \leq (1-\epsilon)\frac{log(n)}{log(npq)}$ for some $\epsilon > 0$, then $E(CC(\mathbf{A}, p, T, \mathbf{y})_i) = o(n)$ for all $i$.*

*b)If, on the other hand, $T \geq (1+\epsilon)(\frac{log(pnq)}{log(n)})$, then $E(CC(\mathbf{A}, p, T, \mathbf{y})_i) = \Omega(n)$ for all $i$.*

Note that the threshold $T = \frac{log(n)}{log(npq)}$ given above is equal to $\frac{log(n)}{log(pE(\lambda_1))}$, since $E(\lambda_1) = nq$. We also note that the expected diameter of the Erdos-Reyni graph is $\frac{log(n)}{log(nq)}$, which is strictly smaller than the threshold given above.

To prove these theorems, we analyze $E(\mathbf{A}^t)$ for any $t$. Note that $E(\mathbf{A}^t)_{ij}$ is the weighted sum of all paths of length $t$ from $i$ to $j$, with each path $\pi$ weighted by $q^{d(\pi)}$, where $d(\pi)$ is the number of distinct edges along the path $\pi$. Note that by symmetry, the off-diagonal entries of $E(\mathbf{A}^t)$ are all the same, as are its diagonal entries; however, the diagonal entries are not necessarily equal to the off-diagonal ones.

We first prove the following lemma to aid our analysis.

**Lemma 1** *Let $i, j, k$ be distinct numbers ranging from $1$ to $n$. Let $Z_{ij,k}(t)$ be the subset of paths of length $t$ from $i$ to $j$ which visit vertex $k$ at some point. Let $z_{ij,k}(t)$ be its weighted sum $\sum_{\pi \in Z_{ij,k}(t)} q^{d(\pi)}$. Then $z_{ij,k} \leq \frac{t-1}{n-2}E(\mathbf{A}^t_{ij})$.*

**Proof 2** *There are $(t-1)$ possible indices to place the vertex $k$. For each fixed index, the weighted sum of all paths with vertex $k$ at that index is $\leq \frac{1}{n-2}E(\mathbf{A}^t_{ij})$, which follows by symmetry with respect to the $n-2$ possible choices of $k$. Combining these factors yields the desired bound.*

We now move on to the estimates of $E(\mathbf{A}^t_{ij})$.

**Lemma 2** *For the purposes of this lemma assume that $\frac{t}{nq} \leq r < \frac{1}{4}$ for some $r$. Then we have*

*a)* $(1-2r)\frac{(nq)^t}{n} \leq E(\mathbf{A}^t)_{ij} \leq (\frac{1}{1-4r})\frac{(nq)^t}{n}$*, if* $i \neq j$ *or if* $i = j$ *and* $t$ *is odd.*

*b)* $(1-2r)\frac{(nq)^t}{n} \leq E(\mathbf{A}^t)_{ii} \leq (\frac{1}{1-4r})(\frac{(nq)^t}{n} + (2nq)^{t/2})$ *if* $t$ *is even.*

**Proof 3** *Let us represent a path by the sequence of the vertices it visits. A path* $\pi$ *of length* $t$ *from* $i$ *to* $j$ *is represented as* $iv_1v_2\cdots v_{t-1}j$*, where* $i$ *and* $j$ *will also be labeled* $v_0$ *and* $v_t$*, respectively.*

*We begin by proving the lower bounds. We have* $E(A^t_{ij}) \geq (n-2)^{t-1}q^t$*. Indeed, there are more than* $(n-2)^{t-1}$ *legitimate paths in* $X_{ij}(t)$ *(under the constraint of no self-edges), and each one has at most* $t$ *distinct edges. Now,* $(n-2)^{t-1} \geq n^{t-1} - 2t(n)^{t-2} = n^{t-1}(1-\frac{2t}{n}) \geq (1-2r)n^{t-1}$ *since* $\frac{t}{n} \leq \frac{t}{qn} \leq r$*.*

*Next, we calculate the upper bounds. Suppose that* $t \geq 1$*. Let* $Y_{ij}(t) \subset X_{ij}(t)$ *consist of those paths in which edges are never repeated immediately, that is,* $v_l \neq v_{l+2}$ *for any index* $l$*. Let* $y_{ij}(t) = \sum_{\pi \in Y_{ij}(t)} q^{d(\pi)}$ *be its weighted sum. We further partition* $Y_{ij}(t)$ *as follows. For each* $k = 1, \ldots, (t-1)$*, let* $Y_{ij,k}(t) \subset Y_{ij}(t)$ *be the subset of those paths for which* $k$ *is the smallest index such that the edge* $v_{k-1}v_k$ *is not revisited later in the path, and* $v_k \neq j$*. Then* $Y_{ij}(t) = \bigsqcup_{k=0}^{t-1} Y_{ij,k}(t)$*. Let* $y_{ij,k}(t)$ *be the weighted sum of* $Y_{ij,k}(t)$*. Also, let* $y_{diff}(t)$ *and* $y_{same}(t)$ *denote the values of* $y_{ij}(t)$ *in the cases* $i \neq j$ *and* $i = j$*, respectively.*

*We will use the following properties for paths* $\pi \in Y_{ij,k}(t)$*. Given* $\pi$*, let* $\pi' \in Y_{v_kj}(t-k)$ *be the truncated path* $v_k, \ldots, v_{t-1}, j$*. We note that* $\pi$ *has at least one edge that* $\pi'$ *does not, namely* $v_{k-1}v_k$*, by definition of* $k$*. Thus* $d(\pi) \geq d(\pi') + 1$*. Furthermore, we note that every node* $v_1, \ldots, v_{k-1}$ *must be present in* $\pi'$*. Indeed, for each such vertex* $v$*, consider the greatest index* $l$ *such that* $v_l = v$*. If* $l < k$*, then, by definition of* $k$*, that means either* $v_l = j$*, in which case it appears in* $\pi'$*, or the edge* $v_{l-1}v_l$ *reappears later in the path. By assumption that* $\pi \in Y_{ij}(t)$*, this edge cannot be repeated immediately; hence* $v = v_l$ *itself must reappear later, contradicting the description of the index* $l$*. So,* $l \geq k$*, that is,* $v$ *indeed appears in* $\pi$*'.*

*These observations imply the following bound:*

$$y_{ij,k}(t) \leq t^{k-1}nqy_{diff}(t-k) \tag{1}$$

*Indeed, to specify a path in $Y_{ij,k}(t)$, we first choose $v_k$ from among $\leq n$ possibilities. Then we choose the truncated path $\pi'$ as described above from $Y_{v_k j}(t-k)$, whose weighted sum is $y_{v_k,j}(t-k)$. Then we choose the $k-1$ vertices $v_1,\ldots,v_{k-1}$. Each of them is repeated in $\pi'$, hence may be chosen from among the $\leq t$ vertices of $\pi'$. Finally, since $d(\pi) \geq d(\pi')+1$, we introduce the additional factor of $q$.*

*Now we focus on the case that $i \neq j$.*

*If $k \geq 1$, we can improve our bound further. Notice that, since $k > 1$, the starting vertex $i$ must appear in the path $\pi'$. So, either $i = v_k$, or $i \neq v_k$. In the former case, we can eliminate a factor of $n$ from (1), and in the latter case, we can introduce a factor of $\frac{t}{n}$ into (1), by Lemma 1 (Note the Lemma applies since $i, j$, and $v_k$ are assumed distinct). We thus obtain the tighter bound*

$$y_{ij,k}(t) \leq t^k q \cdot y_{\mathit{diff}}(t-k) \tag{2}$$

*Now we can prove by induction that $y_{\mathit{diff}}(t) \leq (nq+2)^t$. Indeed, under this inductive hypothesis, the above bounds yield*

$$y_{ij,1}(t) \leq nq(nq+2)^{t-1}$$

*and*

$$\sum_{k=2}^{t-1} y_{ij,k}(t) \leq \sum_{k=2}^{\infty} t^q k(nq+2)^{t-k} \leq t^2 q(nq+2)^{t-2}\frac{1}{1-r}$$
$$\leq 2(nq+2)^{t-1}$$

*Where we used the fact that $(t^2) \leq (nq)^2 \leq n$ and $\frac{1}{1-r} \leq 2$. Combining these bounds together we obtain, as desired, that*

$$y_{ij}(t) = y_{ij,1}(t) + \sum_{k=2}^{t-1} y_{ij,2}(t) \leq (nq+2)(nq+2)^{t-1} = (nq+2)^t$$

*Next, we plug in this bound for $y_{\mathit{diff}}(t)$ into $(1)$, to obtain a bound for $y_{ij}(t)$ (even if $i = j$). We have*

$$y_{ij} \leq \sum_{k=1}^{\infty} t^{k-1}(nq+2)^{t-k+1} \leq \frac{1}{1-r}(nq+2)^t$$

*Now, it is convenient to further bound* $(nq+2)^t \leq \frac{1}{1-2r}(nq)^t$. *Indeed,* $(nq+2)^t = \sum_{k=0}^{t} \binom{t}{k} 2^k n^{t-k} \leq \sum_{k=0}^{\infty}(2t)^k(nq)^{t-k}$, *which is a geometric series with ratio* $\frac{2t}{nq} \leq 2r$, *bounded by* $\frac{1}{1-2r}(nq)^t$.

*Hence, we obtain the following bound on* $y_{ij}(t)$*:*

$$y_{ij}(t) \leq \frac{1}{(1-r)(1-2r)}(nq)^t \leq \frac{1}{1-3r}(nq)^t \tag{3}$$

*We emphasize that this inequality holds only if* $t \geq 1$. *Finally, we extend our analysis from the* $Y_{ij}(t)$ *to all paths. Any arbitrary path from* $i$ *to* $j$ *of length* $t$ *may be obtained by starting with a path in* $Y_{ij}(t-2m)$, *for some* $0 \leq m \leq t/2$ *and performing a sequence of* $m$ *insertions, replacing a vertex* $v$ *with* $vwv$ *instead, for some vertex* $w$. *We obtain the bound*

$$E(\mathbf{A}_{ij}^t) \leq \sum_{m=0}^{\lfloor t/2 \rfloor} y_{ij}(t-2m) \cdot (2nq)^m \tag{4}$$

*Indeed, for each insertion operation, there are two cases: either the inserted vertex* $w$ *is already present in the path, so it can be chosen from among* $\leq t$ *vertices; or it is not already present, in which case it can be chosen from among* $\leq n$ *vertices and introduces a new edge, for an additional factor of* $q$. *Combining the two possibilities, each insertion operation introduces a factor of* $(t + nq) \leq 2nq$.

*To evaluate this sum, we need to consider the two cases outlined in the statement of this lemma.*

*a) Suppose that either* $i \neq j$, *or* $i = j$ *and* $t$ *is odd. In this case, note that the bound (3) can be applied to each* $y_{ij}(t-2m)$, *since if* $t$ *is odd, then* $t - 2m \geq 1$*; and if* $i \neq j$, *we have* $y_{ij}(0) = 0$ *regardless. Combining these bounds with (4), we obtain*

$$E(\mathbf{A}_{ij}^t) \leq \frac{1}{(1-3r)} \sum_{m=0}^{\infty} \frac{1}{n} 2^m (nq)^{t-m} \leq \frac{1}{1-4r} \frac{1}{n}(nq)^t$$

*by a geometric series with ratio* $\frac{2}{nq} < r$. *This completes the proof of part a) of the lemma.*

*b) Now suppose that* $i = j$ *and* $t$ *is even. The sum in (4) can be analyzed in the same way as in a), but with an extra term of* $(2qn)^{t/2}$ *corresponding to the case* $m = \frac{t}{2}$.

We are now ready to prove Theorem 1.

**Proof 4** *By definition,* $E(CC(\mathbf{A},p,T,\mathbf{y})) = E(\sum_{t=0}^{T} p^t \mathbf{A}^t \mathbf{y})$. *By linearity of expectation, this equals* $\sum_{t=0}^{T} p^t E(\mathbf{A}^t)\mathbf{y}$. *Now, for each* $t$ *and each* $i$, *we have* $(E(\mathbf{A}^t)\mathbf{y})_i = \sum_{j=0}^{n} y_j p^t E(\mathbf{A}_{ij}^t)$. *By separating the terms with* $i = j$ *from the terms with* $i \neq j$, *this equals* $n\bar{\mathbf{y}}p^t E(\mathbf{A}_{\textit{diff}}^t) + y_i p^t \cdot (E(\mathbf{A}_{\textit{same}}^t) - E(\mathbf{A}_{\textit{diff}}^t))$, *so we can write*

$$E(CC(\mathbf{A},p,T,\mathbf{y})_i) = \bar{\mathbf{y}}E_1 + y_i E_2$$

*where*

$$E_1 = n\sum_{t=0}^{T} p^t E(\mathbf{A}_{\textit{diff}}^t)$$

*and*

$$E_2 = \sum_{t=0}^{T} p^t \cdot (E(\mathbf{A}_{\textit{same}}^t) - E(\mathbf{A}_{\textit{diff}}^t))$$

*a) By Lemma 2, we know that* $E_1$ *can be bounded*

$$(1-2r)\sum_{t=0}^{T}(npq)^t \leq E_1 \leq \frac{1}{1-4r}\sum_{t=0}^{T}(npq)^t$$

*where* $r = \frac{T}{nq}$. *Since we assume this ratio approaches* $0$, *these bounds imply that indeed* $E_1$ *approaches* $\sum_{t=0}^{T}(npq)^t = \frac{(npq)^{T+1}}{1-npq}$ *as* $n$ *tends to infinity.*

*b) Next, we show that* $E_2 = o(E_1)$. *Indeed, we again use Lemma 2. We have*

$$|E_2| \leq \sum_{t=0}^{T} p^t \cdot (E(\mathbf{A}_{\textit{same}}^t) + E(\mathbf{A}_{\textit{diff}}^t))$$

$$\leq \frac{1}{1-4r}(\sum_{t=0}^{T}(p^t(2nq)^{t/2}) + \frac{(npq)^t}{n})$$

*so the result follows since both terms* $p^t(2nq)^{t/2}$ *and* $\frac{(npq)^t}{n}$ *are lower-order than* $(npq)^t$.

*c) Diffusion centrality is a special case of contextual centrality in which* $\mathbf{y} = \mathbf{1}$, *which has mean* $1$. *The result follows by part a), together with the fact that* $E_1$ *dominates over* $E_2$ *whenever* $\bar{\mathbf{y}} \neq 0$ *by part b).*

Next, we prove Theorem 2.

**Proof 5** *Suppose* $\overline{\mathbf{y}} \neq 0$ *and* $pE(\lambda_1) \geq (1+\epsilon)$ *and that. For Erdos-Renyi graphs,* $E(\lambda_1) = nq$*, so* $pnq \geq (1+\epsilon)$*. In this case, it follows from Theorem 1 that* $E(CC)_i$ *approaches* $\overline{\mathbf{y}}(pnq)^T$*. If* $T \leq (1-\epsilon)\frac{log(n)}{log(npq)}$ *for some* $\epsilon > 0$*, then* $log(|E(CC)_i|) \leq C + log(|(pnq)^T|)$ *for some constant C, which equals* $C + Tlog(pnq) \leq C + (1-\epsilon)log(n)$ *so* $T = O(n^{1-\epsilon}) = o(n)$*. The other direction follows similarly.*

### 1.4 The relationship between contextual centrality and other centrality measures

Degree, eigenvector, Katz, diffusion, and contextual centrality can all be expressed as specific cases of a simple recurrence relation with an intuitive explanation. Roughly speaking, a node's importance in a network can be broken down into two parts: its influence on other nodes in the network through its neighbors, and its individual contribution to the cascade payoff.

Let $\mathbf{c}_t$ be the importance (i.e., centrality) of all nodes in the network at time step $t$. One way to capture the notion of each node's influence on other nodes in the network is through $\mathbf{A}\mathbf{c}_{t-1}$, where $\mathbf{A}$ is the adjacency matrix of the network. This term effectively sums up the importance of the neighbors of each node. With this in mind, we can express $\mathbf{c}_t$ as,

$$\mathbf{c}_t = \alpha \mathbf{A}\mathbf{c}_{t-1} + \boldsymbol{\beta}, \tag{5}$$

where $\alpha$ is a constant and $\boldsymbol{\beta}$ is the individual contribution of each node in the network. It is, of course, possible to parameterize $\alpha$, $\boldsymbol{\beta}$, or $\mathbf{A}$ by $t$ as well, but for simplicity let us assume they remain constant. Expanding this recurrence, we get

$$\mathbf{c}_t = (\alpha\mathbf{A})^t \mathbf{c}_0 + \sum_{i=0}^{t-1} (\alpha\mathbf{A})^i \boldsymbol{\beta}. \tag{6}$$

Now if we substitute $\alpha = p$, $\boldsymbol{\beta} = \mathbf{y}$, and $\mathbf{c}_0 = \mathbf{y}$, then $\mathbf{c}_T$ is exactly equal to CC. Substitutions can be done for all the centrality measures discussed above and are summarized in Table 1.

Contextual centrality is developed upon and generalizes diffusion centrality, but there are two important differences. First, all nodes passed through by the random walk contribute positively and homo-

Table 1: Centrality measures defined by $\mathbf{c}_t = \alpha \mathbf{A} \mathbf{c}_{t-1} + \boldsymbol{\beta}$.

| Centrality | $\alpha$ | $\boldsymbol{\beta}$ | $\mathbf{c}_0$ | $t$ |
|---|---|---|---|---|
| Degree | 1 | $\mathbf{0}$ | $\mathbf{1}$ | 1 |
| Eigenvector | 1 | $\mathbf{0}$ | $\mathbf{1}$ | $\infty$ |
| Katz | $< \frac{1}{\lambda_1}$ | $\mathbf{1}$ | $\mathbf{1}$ | $\infty$ |
| Diffusion | $p$ | $\mathbf{1}$ | $\mathbf{1}$ | $T$ |
| Contextual | $p$ | $\mathbf{y}$ | $\mathbf{y}$ | $T$ |

geneously in diffusion centrality, while the main advantage of contextual centrality is allowing for the heterogeneous contributions. Second, the random walk of contextual centrality starts from the chosen seed, while that of diffusion centrality starts from the neighbors of the chosen seed. Under the condition that $\overline{\mathbf{y}}$ is positive and constant for all entries, contextual centrality inherits the nice nesting properties of diffusion centrality, which encompasses and spans the gap between degree centrality, eigenvector centrality, and Katz centrality. In particular, CC is proportional to degree centrality when $T = 1$, proportional to eigenvector centrality as $T \to \infty$ when $p \geq \lambda_1^{-1}$, and proportional to Katz centrality when $T = \infty$ and $p < \lambda_1^{-1}$. Proof can be found in Banerjee et al.[2].

Contextual centrality is also similar to Katz centrality, but we highlight two crucial differences. First, contextual centrality is more general in that $p$ can be larger than $\lambda_1^{-1}$ and provides essential insights into this region. Second, we allow $T$ to vary according to the specific setting, while in Katz centrality, the diffusion period $T$ is infinite. $T$ carries important implications. For the product that is effective in a short period, such as a coupon that will expire within a day, $T$ is relatively small compared with the diffusion of a new phone, which will be on the market for much longer.

## 1.5 Relationship between approximated cascade payoff and cascade payoff

Contextual centrality aims to maximize objective (4), which provides an approximation to cascade payoff, as in objective (3), by an independent cascade model. In Fig. 1, we analyze the Spearman and Pearson correlation between the two concerning different spreadability. Both correlation measures decrease as

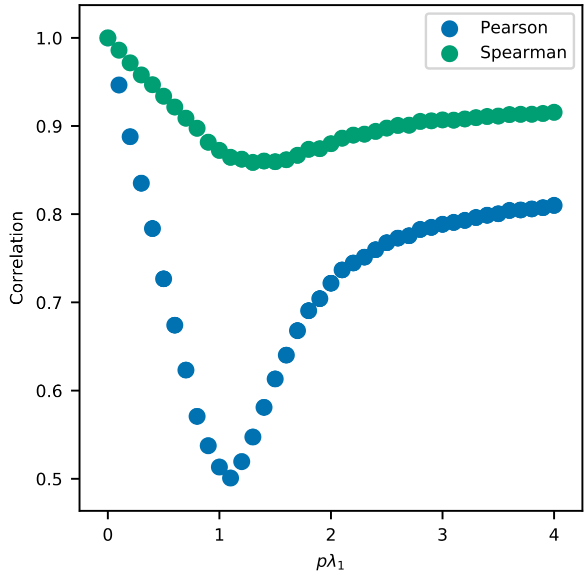


Figure 1: Relationship between approximated cascade payoff and cascade payoff. The y-axis and x-axis display the correlation and the spreadability ( $p\lambda_1$) respectively. Pearson and Spearman's correlation are shown in blue and orange color respectively.

spreadability increases from 0 to 1 and increase afterward. In the bulk part, Spearman's correlation between the two is higher than the Pearson correlation and is around 0.9 or higher. Note that $p\lambda_1 = 1$ is the phase transition in network contagion with the Susceptible-Infected (SI) model and is known as the epidemics threshold[3]. This may explain why we see a different behavior close to $p\lambda_1 = 1$.

## 1.6 Game-theoretic interpretation of contextual centrality with local interactions

Ballester et al. are the first to provide a behavioral foundation for centrality, in particular, Katz-Bonacich centrality, using a complementary linear-quadratic-form network game[4]. They found that one's network position can fully explain the Nash equilibrium in such network games. Similarly, we show that when the spreadability is smaller than one, and agents can interact for an infinite time period, their activity levels can be explained by both their structural positions, as well as their marginal benefits of actions (which corresponds to nodes' contributions).

In the setup of Ballester et al., agents choose actions optimally in response to their neighbors[4]. The

quadratic functional form implies that the utility of individual $i$, $(u_i)$, is quadratic in $i$'s action level $(a_i)$, are dependent on $i$'s neighbors effort, and has a homogeneous marginal benefit $\alpha$ across the population, $u_i = \alpha a_i - \frac{1}{2}a_i^2 + \beta \sum_{j=1}^{n} A_{ij} a_i a_j$, where $\alpha$ is a scalar and $\alpha > 0$. Taking the first-order condition, it is easy to prove that the strategy in Nash equilibrium is $\mathbf{a} = \left(\mathbf{I} - \beta\mathbf{A}\right)^{-1}\alpha \propto c_{\text{Katz}}$, which is proportional to the Katz centrality.

In the previous setup, Ballester et al. assume that the marginal benefit is homogeneous and positive. We relax this constraint, allowing it to vary across individuals $(y_i)$ with and can take on negative values. With this, suppose agent $i$ chooses an action $(a_i)$ according to the following utility function,

$$u_i = a_i y_i - \frac{1}{2}a_i^2 + \beta \sum_{j=1}^{n} A_{ij} a_i a_j. \tag{7}$$

With this variant, the equilibrium strategy becomes,

$$\mathbf{a} = \left(\mathbf{I} - \beta\mathbf{A}\right)^{-1}\mathbf{y}. \tag{8}$$

Eq. (8) has the exact same form as CC when $T \to \infty$, $\beta\lambda_1 < 1$ and $\beta = p$. Hence, we see that contextual centrality approximates agents' equilibrium actions with heterogeneous marginal utilities in this condition.

## 1.7 Differences between contextual centrality and centrality measures developed on weighted networks

There have been some studies that generalize centrality measures to weighted or signed networks. They focus on settings where edge weights represent the strength or the trustiness (a friend or a foe) of the social relationships. The network information captured by these centrality measures can be regarded as a special case of a weighted version of contextual centrality, where $\mathbf{A}$ is a weighted matrix, $p = 1$ and $\bar{\mathbf{y}} = \mathbf{1}$. Weights on network links emphasize social relationships but do not capture the heterogeneous contributions of the nodes - exogenous to the network structure - directly to the cascade payoff. Weighted links and weighted nodes characterize different network dynamics and diffusion objectives. Let us provide a simple illustrative example to explain the differences better. Imagine a network with two disconnected

communities, where one component consists of positive links, and the other consists of negative links. Centrality developed on the weighted or signed network will rank the most-connected node as the top in the community with positive edges (i.e., individuals who all trust one another). However, for a particular marketing campaign, if all individuals in the positive community do not like the product, seeding any individuals in the positive community will hurt the campaign.

For readers' reference, we provide an overview of centrality measures on weighted and signed networks. There are two main strands of work in this literature. First, some studies define new notions of the shortest path that take the weights of the links into account. There are multiple types of modifications: (1) take the inverse of the tie strengths as the shortest path lengths[5,6], (2) using a tuning parameter to trade-off tie strengths and the number of ties[7], (3) adding a temporal aspect to links to minimize the temporal latency[8]. With these new notions, researchers extend existing path-based centrality measures.[5] and[6] extended closeness centrality and betweenness centrality to define the shortest path algorithm to be the least costly path with cost depending solely on tie weights. Opsahl proposes a centrality measure with a generalized degree and shorted paths computation by adding a tuning parameter on tie strengths. Another strand of studies focused on the flow and diffusion processes[7]. Kunegis et al. develop a signed centrality measure using the left eigenvector of the signed network as a generalization of the eigenvector centrality with weighted edges[9]. Other studies develop algorithm-based ranking methods, extending PageRank or HITS. Shahriari and Jalili compute the difference between the scores using PageRank or HITS algorithms for networks consisting of positive and negative links, respectively, as the new measure[10].

## 2 Supplementary figures for empirical analysis

### 2.1 Predictive power of contextual centrality in eventual adoptions

Here we include the supplementary results to examine the robustness of the predictive power of contextual centrality in the eventual adoption outcomes similar to Fig. **??**. We extend the linear regression models to (1) without controlling for village size (as shown in Fig. 2), and (2) with additional controls (as shown in Fig. 3).

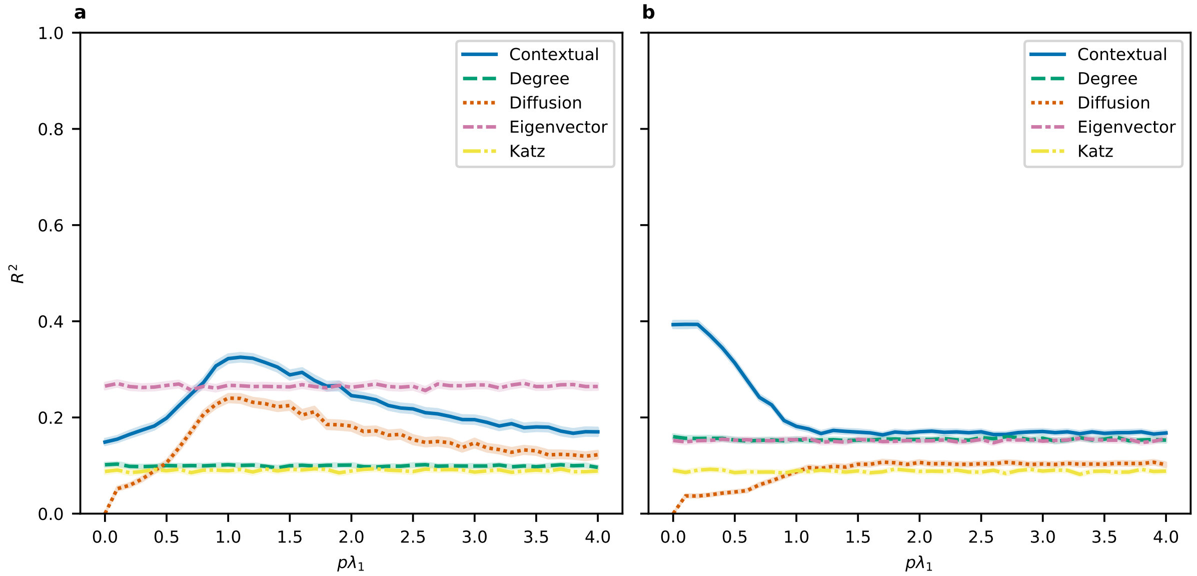


Figure 2: Predictive power of contextual centrality without any controls for (a) microfinance and (b) weather insurance. The y-axis shows the 95% confidence interval of $R^2$ computed from 1000 bootstrap samples from ordinary least squares regressions controlling for village size. The x-axis shows varying values for $p\lambda_1$, which influences only diffusion centrality and contextual centrality.

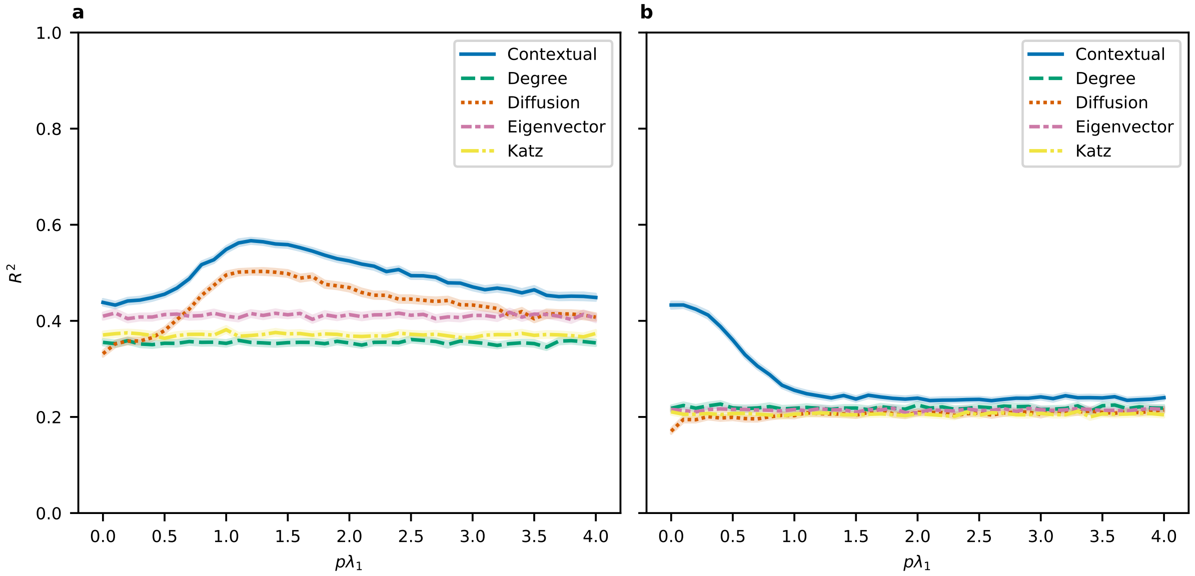


Figure 3: Predictive power of contextual centrality with additional controls for (a) microfinance and (b) weather insurance. For (a), we use village size, savings, self-help group participation, fraction of general caste members, and the fraction of village that is first-informed as done in[11]. For (b), we use village size, number of first-informed households, and fraction of village that is first-informed. The y-axis shows the 95% confidence interval of $R^2$ computed from 1000 bootstrap samples from ordinary least squares regressions controlling for village size. The x-axis shows varying values for $p\lambda_1$, which influences only diffusion centrality and contextual centrality.

## 2.2 Performance relative to other centrality measures on random networks

Here we show supplementary results corresponding to Fig. **??** and Fig. **??**. From Fig. 4 to Fig. 12, we vary the standardized average contribution from -4 to 4. Note in all cases CC has an advantage over other seeding methods when the $p\lambda_1$ is small and loses some of this advantage as $p\lambda_1$ increases. The rate at which CC loses its advantage increases as the magnitude of the standardized average contribution increases. When $p\lambda_1$ is large CC performs comparably to other centrality measures, but in some cases still maintains an advantage.

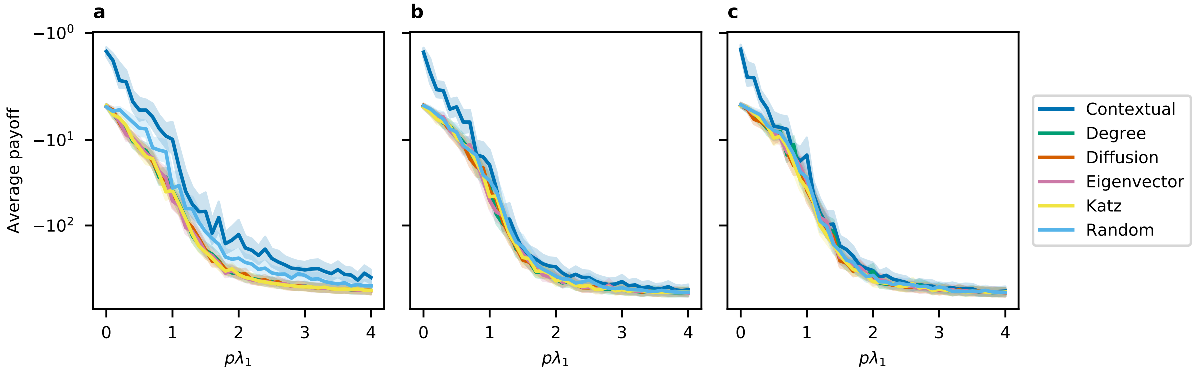


Figure 4: Average payoffs with 95% confidence interval when standardized average contribution is -4 for (a) Barabasi-Albert, (b) Erdos-Renyi, and (c) Watts-Strogatz models.

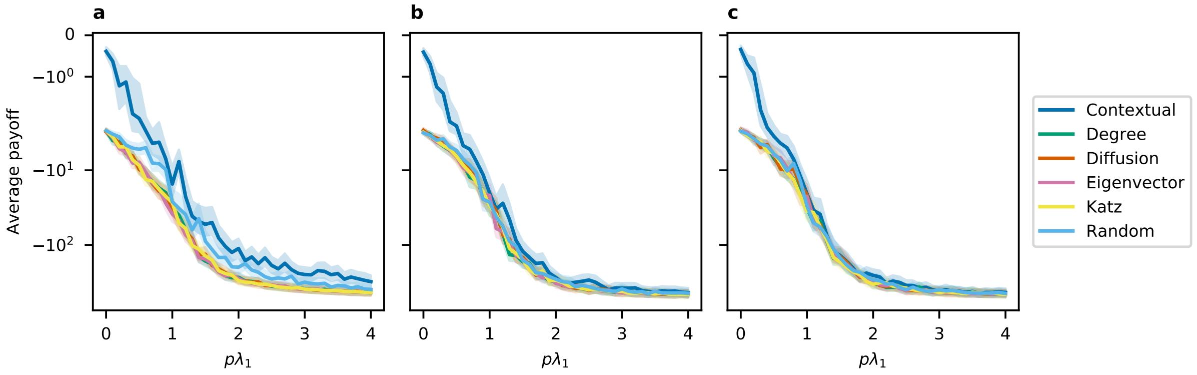


Figure 5: Average payoffs with 95% confidence interval when standardized average contribution is -3 for (a) Barabasi-Albert, (b) Erdos-Renyi, and (c) Watts-Strogatz models.

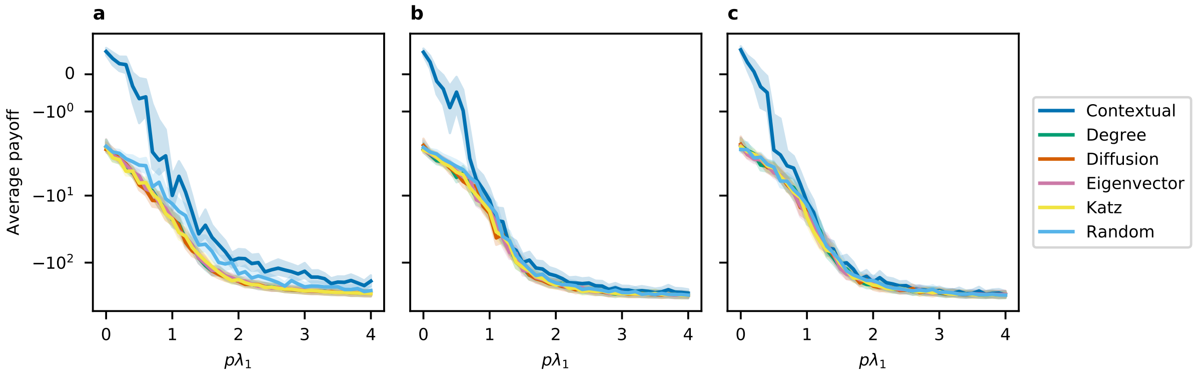


Figure 6: Average payoffs with 95% confidence interval when standardized average contribution is -2 for (a) Barabasi-Albert, (b) Erdos-Renyi, and (c) Watts-Strogatz models.

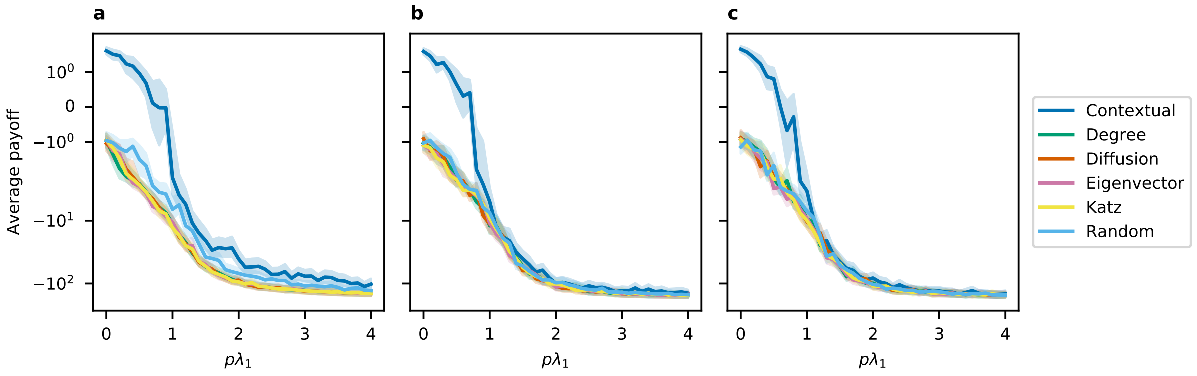


Figure 7: Average payoffs with 95% confidence interval when standardized average contribution is -1 for (a) Barabasi-Albert, (b) Erdos-Renyi, and (c) Watts-Strogatz models.

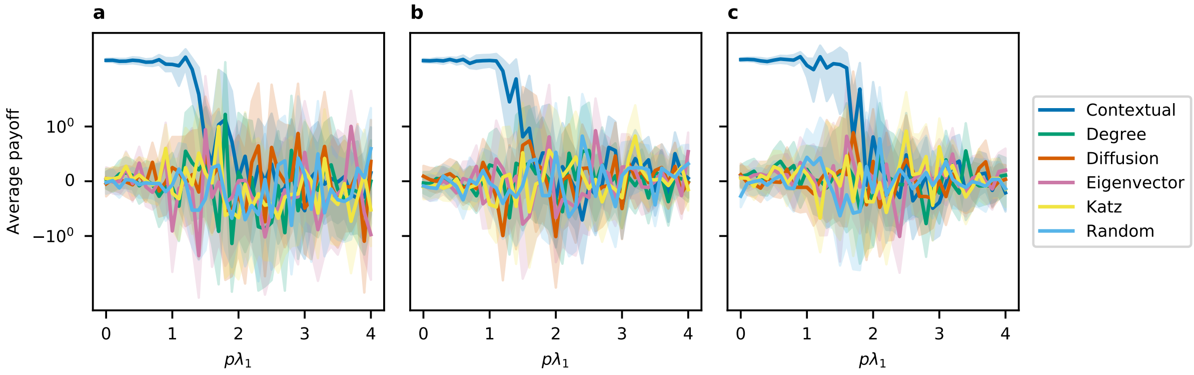


Figure 8: Average payoffs with 95% confidence interval when standardized average contribution is 0 for (a) Barabasi-Albert, (b) Erdos-Renyi, and (c) Watts-Strogatz models.

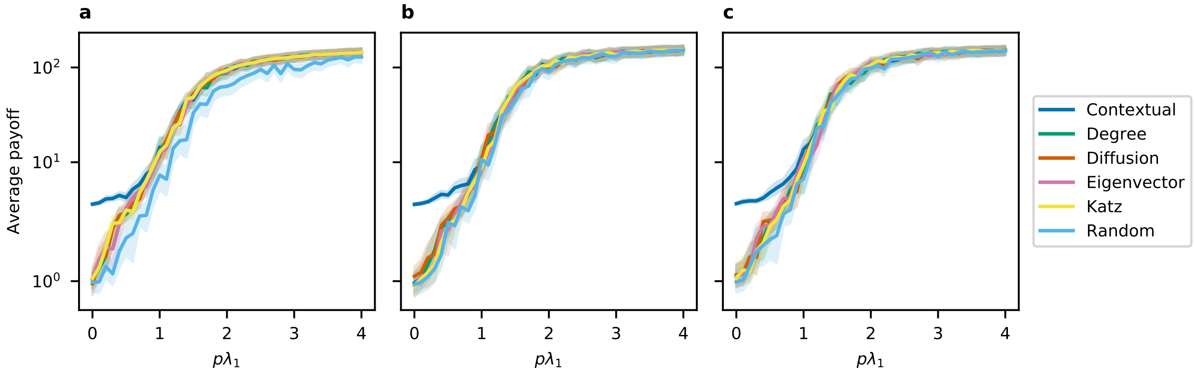


Figure 9: Average payoffs with 95% confidence interval when standardized average contribution is 1 for (a) Barabasi-Albert, (b) Erdos-Renyi, and (c) Watts-Strogatz models.

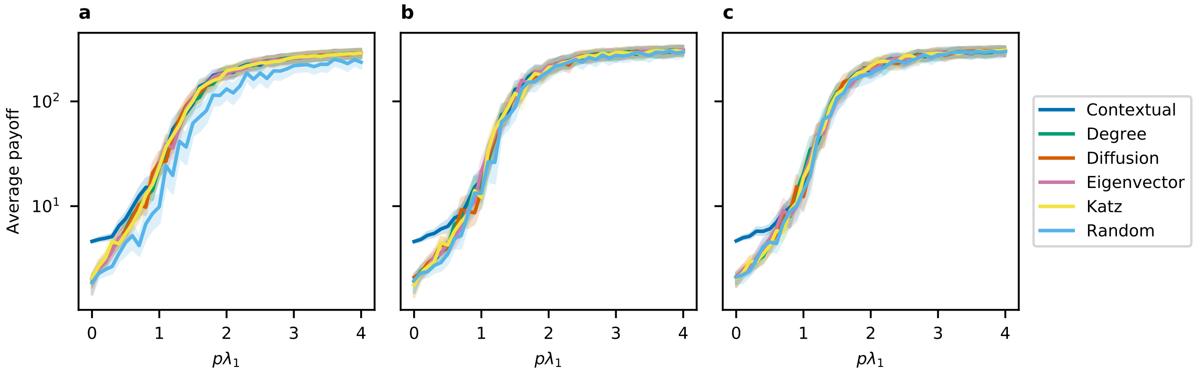


Figure 10: Average payoffs with 95% confidence interval when standardized average contribution is 2 for (a) Barabasi-Albert, (b) Erdos-Renyi, and (c) Watts-Strogatz models.

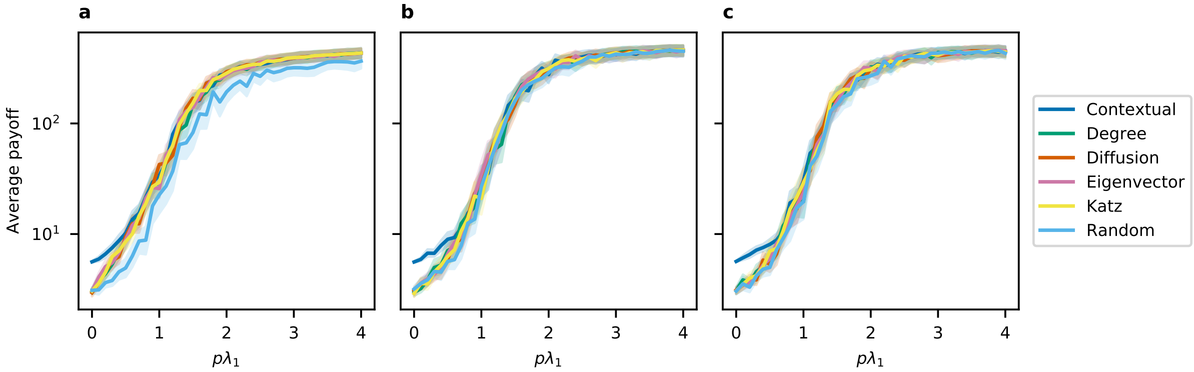


Figure 11: Average payoffs with 95% confidence interval when standardized average contribution is 3 for (a) Barabasi-Albert, (b) Erdos-Renyi, and (c) Watts-Strogatz models.

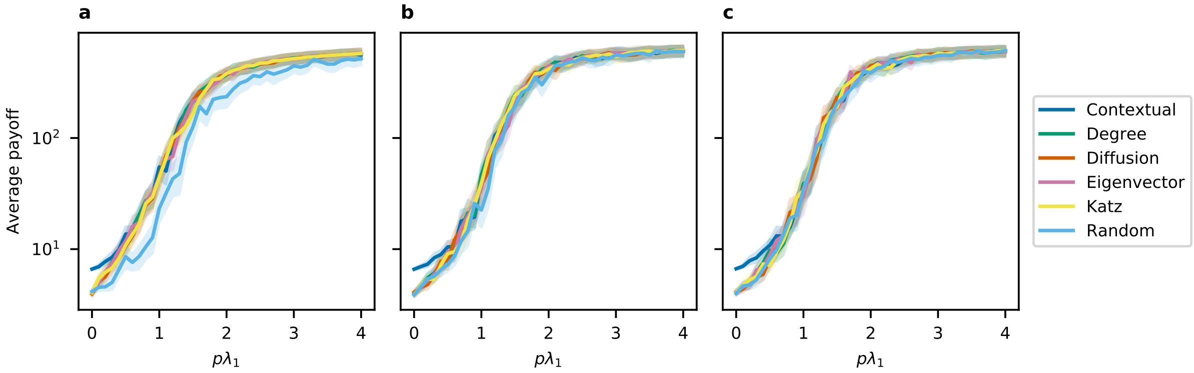


Figure 12: Average payoffs with 95% confidence interval when standardized average contribution is 4 for (a) Barabasi-Albert, (b) Erdos-Renyi, and (c) Watts-Strogatz models.

## 2.3 Average approximated cascade payoff for contextual centrality and the variations of other centrality measures

Here we present the average approximated cascade payoff for contextual centrality and the variations of other centrality measures, including degree centrality (as shown in Fig. 13), diffusion centrality (as shown in Fig. 14), and Katz centrality (as shown in Fig. 15). Note that the approximation does not hold for degree centrality when $p\lambda_1 > 1$ and $T$ is large. However, scaling degree centrality with primary contribution still improves the performance, so we present it here.

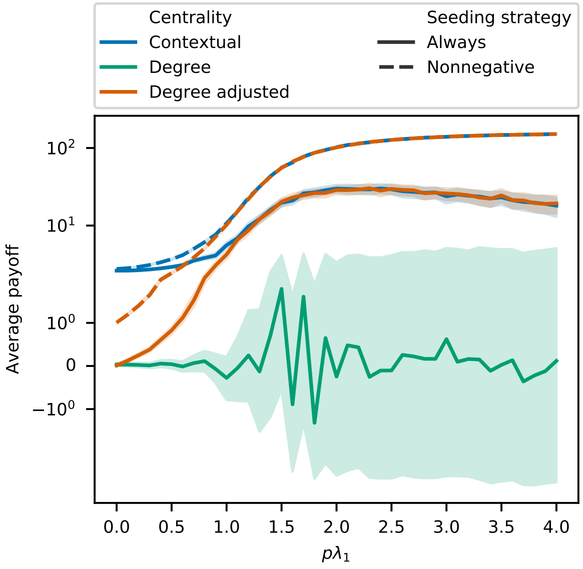


Figure 13: Average cascade payoff for variations of contextual centrality and degree centrality.

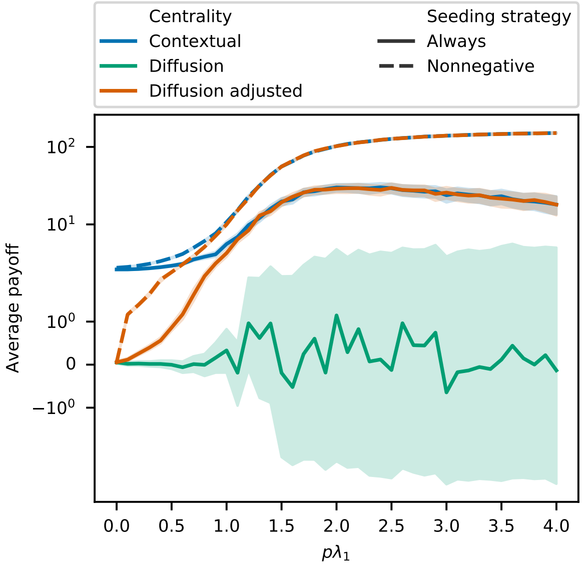


Figure 14: Average cascade payoff for variations of contextual centrality and diffusion centrality.

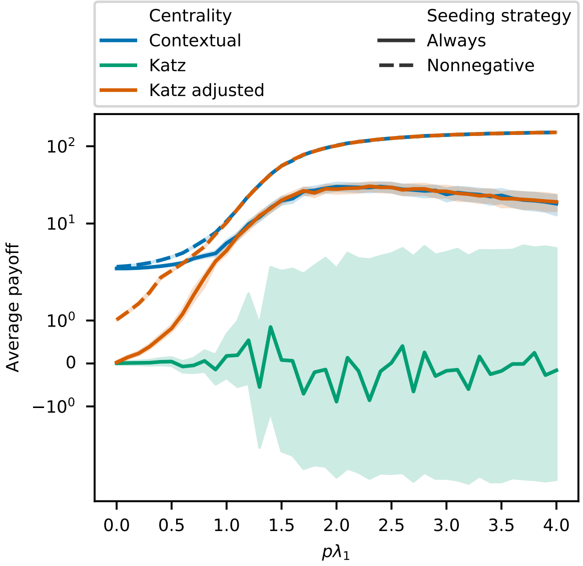


Figure 15: Average cascade payoff for variations of contextual centrality and katz centrality.

### 2.4 Comparison of seeding strategies when $\overline{\mathbf{y}}(\mathbf{U}_1^T\mathbf{y}) < 0$

Here we show the effect of using different seeding strategies on the average cascade payoff. For this plot, we generated 1000 random networks for each graph type (Barabasi-Albert, Erdos-Renyi, and Watts-Strogatz) as before with contributions sampled from a standard normal distribution for (a) continuous and sampled from $\{-1, 1\}$ with equal probability for (b) discrete. We redistributed the contributions to make the signs of $\overline{\mathbf{y}}$ and $\mathbf{U}_1^T\mathbf{y}$ differ if possible, and then filtered out results for which the signs did not differ. More specifically, if the average contribution was negative, the individual with the largest eigenvector centrality score was given the most positive contribution, the individual with the second-largest eigenvector centrality score was given the second most positive contribution, and so on. We used an analogous procedure if the average contribution was positive. Fig. 16 shows that seeding according to the contextual centrality score tends to perform the best as long a $p\lambda_1$ is not too large, after which seeding according to the average contribution performs the best. For small values $p\lambda_1$, seeding always performs as well as, if not better than, seeding according to contextual centrality. As suggested by Eq. (7), seeding according to the primary contribution yields similar results as seeding according to contextual centrality score as $p\lambda_1$ grows large.

## References


1. Banerjee, A., Chandrasekhar, A. G., Duflo, E. & Jackson, M. O. Gossip: Identifying central individuals in a social network. Tech. Rep., National Bureau of Economic Research (2014).

2. Banerjee, A. V., Chandrasekhar, A. G., Duflo, E. & Jackson, M. O. Using gossips to spread information: Theory and evidence from two randomized controlled trials (2017).

3. Chakrabarti, D., Wang, Y., Wang, C., Leskovec, J. & Faloutsos, C. Epidemic thresholds in real networks. *ACM Transactions on Information and System Security (TISSEC)* **10**, 1 (2008).

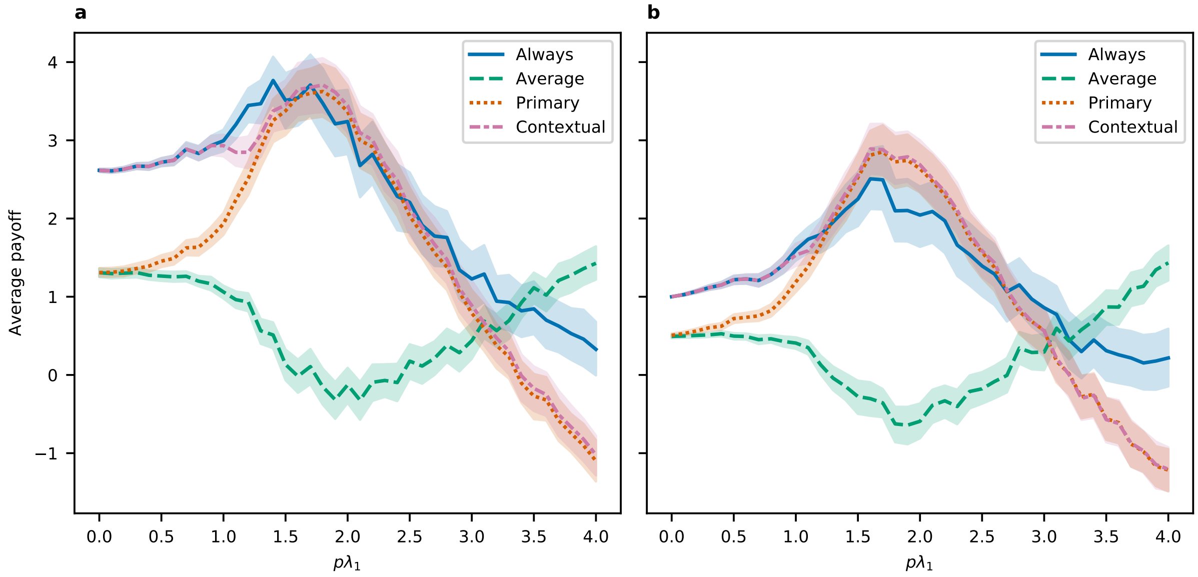


Figure 16: Comparison of seeding strategies when $\overline{\mathbf{y}}(\mathbf{U}_1^T\mathbf{y}) < 0$ for (a) continuous and (b) discrete.

4. Ballester, C., Calvó-Armengol, A. & Zenou, Y. Who's who in networks. wanted: The key player. *Econometrica* **74**, 1403–1417 (2006).

5. Newman, M. E. Scientific collaboration networks. ii. shortest paths, weighted networks, and centrality. *Physical review E* **64**, 016132 (2001).

6. Brandes, U. On variants of shortest-path betweenness centrality and their generic computation. *Social Networks* **30**, 136–145 (2008).

7. Opsahl, T., Agneessens, F. & Skvoretz, J. Node centrality in weighted networks: Generalizing degree and shortest paths. *Social networks* **32**, 245–251 (2010).

8. Williams, M. J. & Musolesi, M. Spatio-temporal networks: reachability, centrality and robustness. *Royal Society open science* **3**, 160196 (2016).

9. Kunegis, J., Lommatzsch, A. & Bauckhage, C. The slashdot zoo: mining a social network with

negative edges. In *Proceedings of the 18th international conference on World wide web*, 741–750 (ACM, 2009).

10. Shahriari, M. & Jalili, M. Ranking nodes in signed social networks. *Social Network Analysis and Mining* **4**, 172 (2014).

11. Banerjee, A., Chandrasekhar, A. G., Duflo, E. & Jackson, M. O. The diffusion of microfinance. *Science* **341**, 1236498 (2013).
